# Cobalt-Catalyzed Reduction of Aldehydes to Alcohols via the Hydroboration Reaction

**DOI:** 10.3390/ijms25147894

**Published:** 2024-07-19

**Authors:** Dariusz Lewandowski, Grzegorz Hreczycho

**Affiliations:** Faculty of Chemistry, Adam Mickiewicz University in Poznań, Uniwersytetu Poznańskiego St. 8, 61-614 Poznan, Poland; dariusz.lewandowski@amu.edu.pl

**Keywords:** aldehydes, reduction, alcohols, cobalt-catalyzed, pinacolborane

## Abstract

A method for the reduction of aldehydes with pinacolborane catalyzed by pincer cobalt complexes based on a triazine backbone is developed in this paper. The presented methodology allows for the transformation of several aldehydes bearing a wide range of electron-withdrawing and electron-donating groups under mild conditions. The presented procedure allows for the direct one-step hydrolysis of the obtained intermediates to the corresponding primary alcohols. A plausible reaction mechanism is proposed.

## 1. Introduction

Alcohols represent one of the major groups of organic chemistry compounds and receive constant attention from both academia and industry. Due to their properties, they are crucial reagents used in chemistry, with applications from solvents to highly specialized drugs or materials [1,2,3,4,5,6]. Each year sees numerous articles devoted to methods for the synthesis and functionalization of alcohols being published [7,8,9,10,11,12].

**Figure 1:**
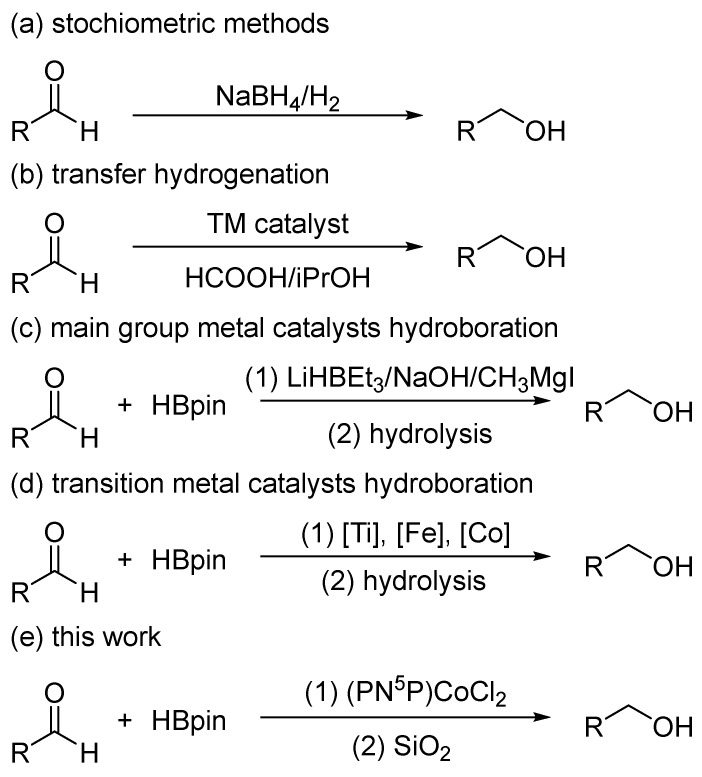
Various methods for the aldehyde reduction.

One of the most important methods for obtaining alcohols is the reduction of carbonyl compounds (aldehydes, ketones) [13]. The most commonly used for this purpose are highly reactive and dangerous hydrides [14] or hydrogen gas [15,16,17,18], which exhibit low selectivity and require much attention being paid to storage and handling (Figure 1a). With this in mind, several alternative reduction methods based on transfer hydrogenation [19,20] (Figure 1b) or hydroelementation [21,22,23] reactions of carbonyl compounds have been developed, employing much safer reagents (formic acid, isopropanol, silanes, and pinacolborane). In the case of hydroelementation, this reaction leads to intermediate silyl ethers or borate esters, which, upon hydrolysis, yield the corresponding alcohol. In this context, methods based on pinacolborane hydroboration reactions appear to be of particular interest, which, unlike hydrosilylation reactions, most often do not require the use of expensive precious metal catalysts [24].

Although the hydroboration of aldehydes and their subsequent hydrolysis to primary alcohols are known to occur without a catalyst, this process has some limitations [25]. In particular, it requires elevated temperatures or long reaction times and the elimination of the solvents, which leads to low conversion for most solid substrates. For this reason, in the scientific literature, there are many examples utilizing catalysts based on main- or transition-group elements [21,23,26].

Several s-block metals have proven to be effective catalysts for aldehyde hydroboration reactions (Figure 1c) [21,23]. Unfortunately, the catalysis with LiHBEt_3_ [27], nBuLi [28], or NaH [29] involves highly reactive and hazardous compounds, while the catalysis with NaOH [30], tBuONa [31], or CH_3_MgI [32] may lead to side reactions, resulting in low process selectivity. In the case of transition metals, there are known examples using heterogeneous catalysis based on Ti [33], Fe [34], and Co [35,36], as well as homogeneous catalysis based on Ti [37], Mn [38], Fe [39], Ni [40], Cu [41], and Zn [42] (Figure 1d). Regarding cobalt catalysts, recent comprehensive research concerning the use of SNS cobalt complexes in the hydroboration reaction of aldehydes has been published [43]. However, to maintain the selectivity of the reactions toward the formyl group, they must be carried out in deuterated benzene; otherwise, the authors observed a partial reduction of the acyl group. There are also reports on the use of Co(IMes)_2_Cl [44] and Co(acac)_3_ [45] complexes, but both of these systems also catalyze the reduction reaction of ketones, preventing the selective reduction of the formyl group for multifunctional compounds.

With this in mind, and encouraged by our previous research on the use of pincer cobalt complexes in catalysis [46,47,48,49,50], we decided to investigate the possibility of using them in aldehyde reduction reactions. The decision to choose pincer complexes was dictated by their high air and thermal stability as well as their catalytic activity in many chemical transformations [51,52]. We have demonstrated their high catalytic activity in the hydroboration reactions of silylacetylenes [53], alkenes [54], and allenes [55], as well as in the reduction reaction of ketones with diphenylsilane [56]. Moreover, a great advantage of the discussed triazine backbone-based ligands is their straightforward synthesis from readily available precursors. Herein, we present a method that allows for the reduction of aldehydes with pinacolborane under mild conditions and showing high tolerance to substrate functional groups (Figure 1e).

## 2. Results and Discussion

**Figure 2:**
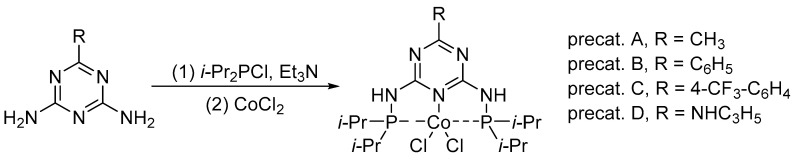
Procedure for the synthesis of pincer cobalt complexes.

First, according to the synthetic protocol developed by the Kempe group [57,58], we synthesized a series of pincer cobalt complexes based on a triazine backbone (Figure 2), which we then tested in a model reduction reaction of 4-methylbenzaldehyde with pinacolborane.

The initial results show the catalytic activity of the obtained precatalysts in the aldehyde hydroboration reaction (Table 1). Among the tested complexes, the highest activity was shown by precatalyst D, which led to full conversion in the reaction carried out at 35 °C for 60 min. Control experiments without a catalyst, with cobalt (II) chloride, or with ligands showed minor conversions, confirming that the obtained complexes are fully responsible for the catalytic activity.

**Figure 3:**
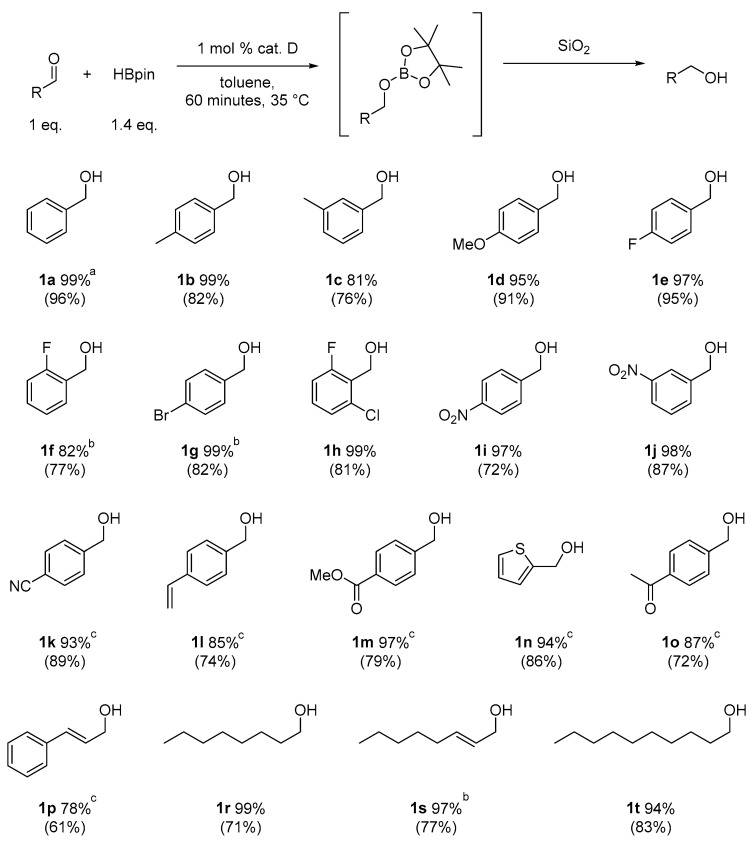
Reduction of aldehydes. ^a^ Conversion of aldehyde determined by GC with n-dodecane as the internal standard—the isolated yields are in parentheses; ^b^ 2 mol% cat. D; ^c^ 5 mol% cat. D.

With optimal conditions, we decided to apply them in the reduction of various aldehydes, omitting the isolation of borate esters by performing hydrolysis to the corresponding alcohols using SiO_2_ (Figure 3). The reaction with benzaldehyde (**1a**) proceeded smoothly, resulting in benzyl alcohol in a quantitative yield. In the case of reactions with aromatic aldehydes with electron-donating groups (**1b**–**1d**) at both para and meta positions, we observed high catalytic activity of the designed system. In the case of halogen-substituted aromatic aldehydes (**1e**–**1h**), we observed very good conversions, although for 2-fluorobenzaldehyde and 4-bromobenzaldehyde, we had to increase the amount of catalyst to 2 mol%. Interestingly, for the strongly deactivating nitro group in the para or meta position (**1i**,**1j**), we observed smooth conversions to the corresponding alcohols. However, for some of the more challenging aldehydes with electron-withdrawing groups (**1k**, **1m**, **1o**), as well as for 4-vinylbenzaldehyde (**1l**) or thiophene-2-carboxaldehyde (**1n**), we had to increase the amount of catalyst to 5 mol%. In the reaction with cinnamaldehyde (**1p**), this system allowed its conversion to cinnamyl alcohol. Similarly, the reaction of (E)-oct-2-enal (**1s**) yielded a selective reduction with retention of the conjugated double bond. In the case of reactions with aliphatic aldehydes (**1r**, **1t**), the reduction proceeded smoothly, producing products with high yields. Importantly, the presented examples demonstrate the high functional group tolerance of the developed method, permitting the selective reduction of the formyl group in the presence of other reactive functional groups (nitro, nitrile, vinyl) without any side reactions. Moreover, the developed method allows for the precise reduction of the formyl group in substituted esters and ketones while preserving their carbonyl group. This selectivity allows the developed procedure to be used as a powerful tool for reducing the formyl group in complex molecules, increasing its application potential.

To confirm the utility of the presented method for the reduction of aldehydes, we performed comparative tests under the developed conditions with and without a catalyst (Table 2). In each of the carried-out reactions, the addition of a catalyst proved to be crucial for achieving high conversion rates. This indicates the applicability of the developed method, particularly for substrates that are sensitive to high temperatures or which require the use of a solvent.

**Figure 4:**
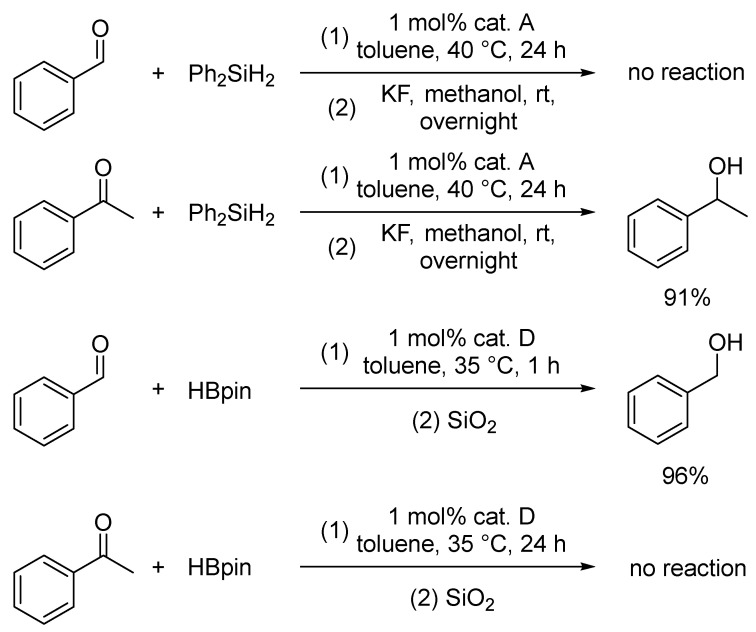
Comparison of cobalt PNP traizine-based methods for the reduction of cabonyl compounds using hydroelementation reactions.

The presented system complements our previously developed method for the reduction of ketones with diphenylsilane (Figure 4) [56]. Interestingly, in the case of the hydrosilylation-based reduction presented there, the developed system only allowed the reduction of ketones and showed no catalytic activity in the reduction of the formyl group. By contrast, we observed the opposite reactivity in the case of the hydroboration-based reaction presented here, in which it is the ketones that are not reduced.

**Figure 5:**
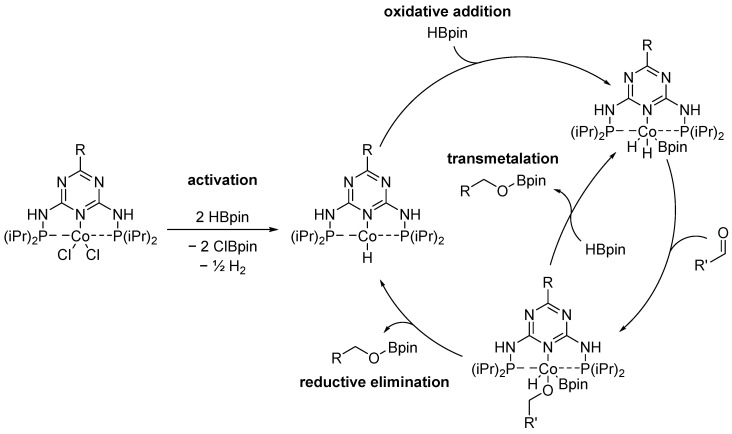
Proposed catalytic cycle.

Based on our previous studies and literature reports [47,48,53,54,55,56,59,60], we assume that the reaction follows a Co(I)/Co(III) mechanism (Figure 5). In the first step, the cobalt precatalyst is activated by pinacolborane, resulting in the active form of the Co(I) catalyst. It then undergoes oxidative addition, resulting in a Co(III) molecule, into which the aldehyde is inserted. In the next step, the product is released by means of reductive elimination with simultaneous Co(I) regeneration. Alternatively, reductive elimination can be replaced by transmetallation with a pinacolborane molecule, resulting in the formation of the product and the regeneration of Co(III).

## 3. Materials and Methods

Air- and moisture-sensitive reactions were carried out under an argon atmosphere using standard Schlenk techniques or a glove box. Solvents used for all experiments were purchased from Honeywell (Charlotte, NC, USA) or Sigma Aldrich (St. Louis, MO, USA) (Merck, Rahway, NJ, USA), dried over calcium hydride (CaH_2_), and purified by means of distillation. Toluene was additionally dried over sodium. Ligands and Co complexes were prepared in accordance with previously reported methods [61] using reagents purchased from Sigma Aldrich (Merck) or ABCR GmBH (Karlsruhe, Germany). Pinacolborane and aldehydes were purchased from Sigma-Aldrich, dried over calcium hydride, and purified by means of distillation. The progress of reactions (conversion of aldehyde) was monitored via GC chromatography using Agilent 8860 GC and Agilent 5977B GC/MSD with the Agilent 8860 GC System (Agilent, Santa Clara, CA, USA). The structures of products were determined by means of NMR spectroscopy and mass spectrometry. The ^1^H NMR (400 MHz), ^13^C NMR (101 MHz), and ^31^P NMR (162 MHz) spectra were recorded on a Bruker Avance III HD NanoBay spectrometer (Bruker, Billerica, MA, USA), using chloroform-d1 (CDCl_3_) as a solvent. Deuterated solvents were purchased from Deutero GmbH (Kastellaun, Germany) (CDCl_3_ 99.6 atom% D) or Sigma Aldrich (Merck) (CDCl_3_ 99.8 atom% D) and were used as received.

### General Procedure for the Synthesis of Compounds (***1a***–***1t***)

To a 12 mL vial equipped with a magnetic stirring bar, precatalyst E (0.01 eq. for **1a**-**1e**, **1h**–**1j**, **1r**, **1t** or 0.02 eq. for **1f**, **1g**, **1s** or 0.05 eq. for **1k**–**1p**), toluene (170 μL), the corresponding aldehyde (0.3 mmol, 1.0 eq.), and pinacolborane (0.45 mmol, 1.4 eq.) were added under an inert gas atmosphere (glove box). A reference sample was taken. Subsequently, the reaction mixture was stirred at 35 °C for 60 min, and the progress of the reaction was monitored by GC or GC/MS. After the reaction was completed, the volatiles were evaporated under reduced pressure. The residue mixture was subjected to column chromatography using silica gel and hexane/ethyl acetate (1:1, *v*/*v*) as an eluent, which was then evaporated under reduced pressure giving the desired product. The products were identified by means of ^1^H and ^13^C spectroscopies and mass spectrometry.

## 4. Conclusions

In summary, we have developed a catalytic method for the reduction of aldehydes to the corresponding alcohols under mild conditions utilizing cobalt pincer complexes based on a triazine backbone. Aldehydes possessing a wide range of electron-withdrawing and electron-donating groups were reduced. The presented protocol demonstrates high tolerance to the functional groups of the substrates, allowing for the selective reduction of the formyl group in the presence of other reactive functional groups. Moreover, the simple work-up of the reaction is a significant advantage of the presented method, with one-pot hydrolysis of the intermediate to the corresponding alcohols. Based on our earlier studies, we also present a possible reaction mechanism.

## Figures and Tables

**Table 1:** Optimization for the cobalt-catalyzed reduction of aldehydes.

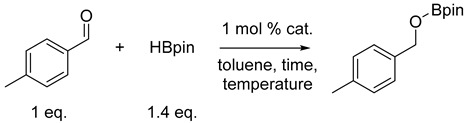				
No.	Catalyst	Time	Temperature	Conversion of Aldehyde ^1^
1	precat. A	10 min	25 °C	15%
2	precat. A	30 min	25 °C	21%
3	precat. A	30 min	35 °C	54%
4	precat. A	60 min	35 °C	72%
5	precat. B	60 min	35 °C	32%
6	precat. C	60 min	35 °C	17%
7	precat. D	60 min	35 °C	99%
8	-	60 min	35 °C	11%
9	CoCl_2_	60 min	35 °C	14%
10	ligand A	60 min	35 °C	0%
11	ligand B	60 min	35 °C	2%
12	ligand C	60 min	35 °C	3%
13	ligand D	60 min	35 °C	3%

^1^ Conversion of aldehyde determined by GC with n-dodecane as the internal standard.

**Table 2:** Comparison of aldehyde reduction under catalytic and catalyst-free conditions.

Aldehyde	Conversion in Catalyst-Free Conditions ^1^	Conversion with Addition of Catalyst ^2^
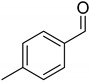	11%	99%
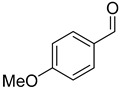	2%	95%
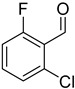	10%	99%
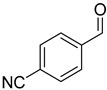	<1%	93% ^3^
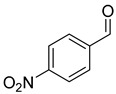	6%	97%
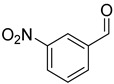	2%	98%
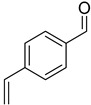	2%	85% ^3^
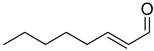	8%	97% ^4^

^1^ Aldehyde/borane ratio of 1:1.5, 35 °C, 60 min, toluene; ^2^ Aldehyde/borane ratio of 1:1.4, 1 mol% cat. D, 35 °C, 60 min, toluene; ^3^ 5 mol% cat. D; ^4^ 2 mol% cat. D.

## Data Availability

All data generated or analyzed during this study are included in this published article (and its Supplementary Information files: Table S1 and S2, Figures S1–S38).

## Supplementary Materials

The following supporting information can be downloaded at: https://www.mdpi.com/1422-0067/25/14/7894/s1. References [61,62,63,64,65,66,67,68,69,70] are cited in the supplementary materials.
