# Supplementary material for: Cobalt-Catalyzed Reduction of Aldehydes to Alcohols via the Hydroboration Reaction
Source: Int J Mol Sci. 2024 Jul 19;25(14):7894. doi: 10.3390/ijms25147894 (PMC11487440; doi:10.3390/ijms25147894)
Supplement: Supplementary file 1 [file ijms-25-07894-s001.zip › ijms-3102874-supplementary.pdf]

Cobalt-catalyzed reduction of aldehydes to alcohols via hydroboration reaction

Dariusz Lewandowski[a] and Grzegorz Hreczycho*[a]

[a] - Faculty of Chemistry, Adam Mickiewicz University in Poznań; Uniwersytetu Poznańskiego St. 8, 61-614 Poznań (Poland). *E-mail: g.h@amu.edu.pl

Table of contents

1. General information 2
2. Optimization studies 3
3. Experimental procedures 4
   General procedure for ligand preparation 4
   General procedure for Co-complex preparation 4
   General procedure for the synthesis of compounds 1a-1t 4
4. Characterization data for all products 5
5. Spectra for all products 9
6. References 28

1. General information

Air- and moisture sensitive reactions were carried out under an argon atmosphere using standard Schlenk techniques or a glove box. Solvents used for all experiments were purchased from Honeywell or Sigma Aldrich (Merck), dried over calcium hydride (CaH_2), and purified by distillation. Toluene was additionally dried over sodium. Ligands and Co-complexes were prepared in accordance with previously reported methods,[1] using reagents purchased from Sigma Aldrich (Merck) or ABCR GmBH. Pinacolborane and aldehydes were purchased from Sigma-Aldrich, dried over calcium hydride and purified by distillation. The progress of reactions (conversion of aldehyde) was monitored by GC chromatography using Agilent 8860 GC and Agilent 5977B GC/MSD with Agilent 8860 GC System. The structures of products were determined by NMR spectroscopy and mass spectrometry. The ^{1}H NMR (400 MHz), ^{13}C NMR (101 MHz), and ^{31}P NMR (162 MHz) spectra were recorded on a Bruker Avance III HD NanoBay spectrometer, using chloroform-d1 ($CDCl_3$) as a solvent. Deuterated solvents were purchased from Deutero GmbH ($CDCl_3$ 99.6 atom% D) or Sigma Aldrich (Merck) ($CDCl_3$ 99.8 atom% D) and used as received.

2. Optimization studies

Table S1. Structures of obtained complexes.

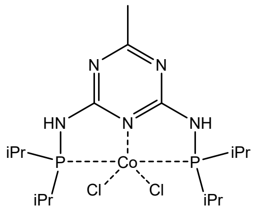<br>precat. A	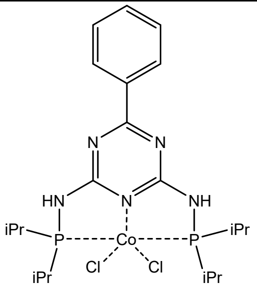<br>precat. B
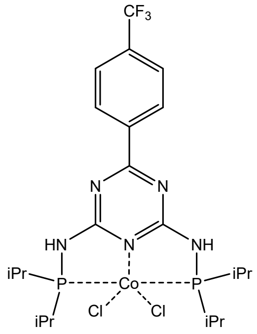<br>precat. C	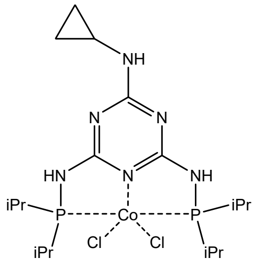<br>precat. D

Table S2. Optimization for cobalt catalyzed reduction of aldehydes.

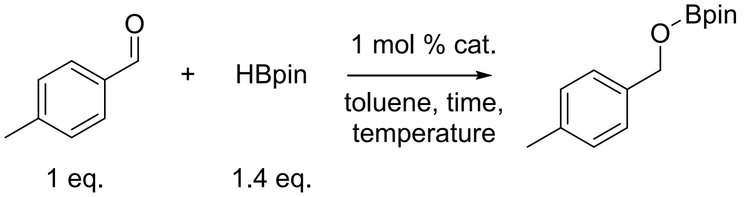

No.	Catalyst	Time	Temperature	Conversion of aldehyde[a]
1	precat. A	10 min	25°C	15%
2	precat. A	30 min	25°C	21%
3	precat. A	30 min	35°C	54%
4	precat. A	60 min	35°C	72%
5	precat. B	60 min	35°C	32%
6	precat. C	60 min	35°C	17%
7	precat. D	60 min	35°C	99%
8	-	60 min	35°C	11%
9	$CoCl_2$	60 min	35°C	14%

a - Conversion of aldehyde determined by GC with n-dodecane as the internal standard.

3. Experimental procedures

General procedure for ligand preparation

Ligands were prepared according to the previously reported method.[1] To a 250 mL Schlenk flask equipped with a magnetic stirring bar, corresponding triazine diamine (20 mmol, 1 eq.), triethylamine (80 mmol, 4 eq.), and THF (60 mL) were added under an inert gas atmosphere. The solution was cooled to 0°C and the diisopropylchlorophosphine (42 mmol, 2.1 eq) was added dropwise by syringe. After all the phosphine was added, the reaction mixture was sealed and the reaction was warmed to room temperature and then heated to 60°C overnight. After cooling, triethyl ammonium chloride was allowed to settle and the organic phase was isolated by filtration. The remaining salt was washed with THF once and the combined organic phases were concentrated and dried under a high vacuum giving the PNP ligand, which was then recrystallized from hot toluene.

General procedure for Co-complex preparation

Cobalt complexes were prepared according to the previously reported method.[1] To a 100 mL Schlenk flask equipped with a magnetic stirring bar, $CoCl_2$ (1.4 mmol, 1 eq.), and THF (12 mL) were added under an inert gas atmosphere. The corresponding PNP-ligand (1.4 mmol, 1 eq.) was dissolved in THF (12 mL) and added to the stirred suspension of $CoCl_2$. The Schlenk flask was sealed and heated to 60°C overnight. After this time, the solvent from the reaction mixture was evaporated giving the Co-complex, which was dried under a high vacuum.

General procedure for the synthesis of compounds (1a-1t)

To a 12mL vial equipped with a magnetic stirring bar, precatalyst E (0.01 eq. for 1a-1e, 1h-1j, 1r, 1t or 0.02 eq. for 1f, 1g, 1s or 0.05 eq. for 1k-1p), toluene (170 µL), corresponding aldehyde (0.3 mmol, 1.0 eq.), pinacolborane (0.45 mmol, 1.4 eq.) were added under inert gas atmosphere (glove box). A reference sample was taken. Subsequently, the reaction mixture was stirred at 35°C for 60 minutes, and the progress of the reaction was monitored by GC or GC/MS. Subsequently, the reaction mixture was stirred at 35°C for 60 minutes. After the reaction was completed the volatiles were evaporated under reduced pressure. The residue mixture was subjected to column chromatography using silica gel and hexane:ethyl acetate (1:1, v/v) as an eluent which was then evaporated under reduced pressure giving the desired product. The products were identified by ^{1}H and ^{13}C spectroscopies and mass spectrometry.

4. Characterization data for all products

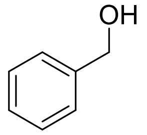

phenylmethanol (1a) was obtained with 96% yield, and is known in the literature. All spectroscopic data are in agreement.[2] ^{1}H NMR (400 MHz, $CDCl_3$, 25°C) δ (ppm): 2.18 (br. s, 1H), 4.57 (s, 2H), 7.16-7.31 (m, 5H). ^{13}C NMR (101 MHz, $CDCl_3$, 25°C) δ (ppm): 65.3, 127.0, 127.7, 128.6, 140.8. EI-MS m/z (rel. int.): 108 (87), 91 (16), 79 (100), 77 (64), 51 (22).

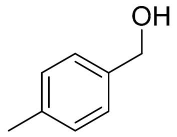

p-tolylmethanol (1b) was obtained with 82% yield, and is known in the literature. All spectroscopic data are in agreement.[3] ^{1}H NMR (400 MHz, $CDCl_3$, 25°C) δ (ppm): 1.94 (br. s, 1H), 2.39 (s, 3H), 4.65 (s, 2H), 7.20 (d, J= 7.8 Hz, 2H), 7.28 (d, J= 7.9 Hz, 2H). ^{13}C NMR (101 MHz, $CDCl_3$, 25°C) δ (ppm): 21.2, 65.2, 127.1, 129.2, 137.4, 137.9. EI-MS m/z (rel. int.): 122 (70), 107 (90), 91 (98), 79 (100), 77 (78), 65 (45), 63 (29), 51 (29).

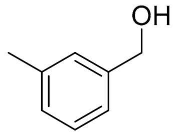

m-tolylmethanol (1c) was obtained with 76% yield, and is known in the literature. All spectroscopic data are in agreement.[4] ^{1}H NMR (400 MHz, $CDCl_3$, 25°C) δ (ppm): 2.41 (s, 3H), 2.65 (br. s, 1H), 4.63 (s, 2H), 7.17 (dd, J= 14.3, 7.6 Hz, 2H), 7.20 (s, 1H), 7.29 (t, J = 7.5 Hz, 1H). ^{13}C NMR (101 MHz, $CDCl_3$, 25°C) δ (ppm): 21.4, 65.2, 124.1, 127.8, 128.3, 128.5, 138.2, 140.9. EI-MS m/z (rel. int.): 122 (80), 107 (88), 91 (100), 79 (86), 77 (81), 65 (41), 63 (31), 51 (29).

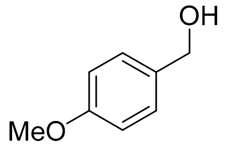

(4-methoxyphenyl)methanol (1d) was obtained with 91% yield, and is known in the literature. All spectroscopic data are in agreement.[3] ^{1}H NMR (400 MHz, $CDCl_3$, 25°C) δ (ppm): 2.06 (br. s, 1H), 3.70 (s, 3H), 4.48 (s, 2H), 6.79 (d, J= 8.6 Hz, 2H), 7.17 (d, J= 8.5 Hz, 2H). ^{13}C NMR (101 MHz, $CDCl_3$, 25°C) δ (ppm): 55.3, 64.9, 113.9, 128.7, 133.2, 159.2. EI-MS m/z (rel. int.): 138 (100), 121 (52), 109 (69), 107 (27), 94 (31), 77 (47), 65 (13), 63 (11), 51 (12).

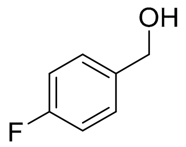

(4-fluorophenyl)methanol (1e) was obtained with 95% yield, and is known in the literature. All spectroscopic data are in agreement.[3] ^{1}H NMR (400 MHz, $CDCl_3$, 25°C) δ (ppm): 2.59 (br. s, 1H), 4.60 (s, 2H), 7.03-7.06 (m, 2H), 7.29-7.32 (m, 2H). ^{13}C NMR (101 MHz, $CDCl_3$, 25°C) δ (ppm): 64.4, 115.3 (d, J= 21.4 Hz), 128.7 (d, J= 8.1 Hz), 136.6 (d, J= 3.3 Hz), 162.3 (d, J= 245.5 Hz). EI-MS m/z (rel. int.): 126 (92), 109 (32), 105 (39), 97 (100), 83 (12), 77 (40), 57 (12), 51 (13).

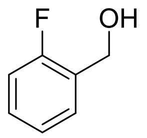

(2-fluorophenyl)methanol (1f) was obtained with 77% yield, and is known in the literature. All spectroscopic data are in agreement.[3] ^{1}H NMR (400 MHz, $CDCl_3$, 25°C) δ (ppm): 2.34

(br. s, 1H), 4.75 (d, J= 5.3 Hz, 2H), 7.05-7.08 (m, 1H), 7.15-7.17 (m, 1H), 7.28-7.31 (m, 1H), 7.42-7.44 (m, 1H). ^{13}C NMR (101 MHz, $CDCl_3$, 25°C) δ (ppm): 59.3 (d, J= 4.4 Hz), 115.2 (d, J= 21.1 Hz), 124.2 (d, J= 3.7 Hz), 127.8 (d, J= 14.5 Hz), 129.3 (d, J= 4.8 Hz), 129.3 (d, J= 8.1 Hz), 160.6 (d, 246.1 Hz). EI-MS m/z (rel. int.): 126 (100), 109 (30), 105 (80), 97 (84), 83 (14), 77 (54), 51 (18).

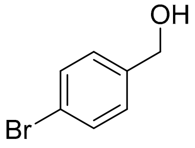

(4-bromophenyl)methanol (1g) was obtained with 82% yield, and is known in the literature. All spectroscopic data are in agreement.[3] ^{1}H NMR (400 MHz, $CDCl_3$, 25°C) δ (ppm): 1.91 (br. s, 1H), 4.56 (s, 2H), 7.15 (d, J= 8.6 Hz, 2H), 7.40 (d, J= 8.4 Hz, 2H). ^{13}C NMR (101 MHz, $CDCl_3$, 25°C) δ (ppm): 64.6, 121.5, 128.6, 131.6, 139.7. EI-MS m/z (rel. int.): 188 (52), 186 (56), 159 (11), 157 (19), 107 (80), 90 (15), 89 (10), 79 (100), 77 (94), 63 (10), 51 (22).

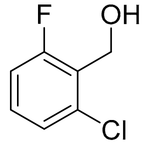

(2-chloro-6-fluorophenyl)methanol (1h) was obtained with 81% yield, and is known in the literature. All spectroscopic data are in agreement.[5] ^{1}H NMR (400 MHz, $CDCl_3$, 25°C) δ (ppm): 1.89 (br. s, 1H), 4.78 (d, J= 1.9 Hz, 2H), 6.92-6.97 (m, 1H), 7.12-7.19 (m, 2H). ^{13}C NMR (101 MHz, $CDCl_3$, 25°C) δ (ppm): 56.2 (d, J= 4.7 Hz), 114.4 (d, J= 22.9 Hz), 125.4 (d, J= 3.6 Hz), 126.1 (d, J= 18.1 Hz), 130.0 (d, J= 9.8 Hz), 135.5 (d, J= 5.5 Hz), 161.6 (d, J= 249.7 Hz). EI-MS m/z (rel. int.): 162 (21), 161 (15), 160 (64), 159 (34), 143 (29), 141 (11), 139 (30), 131 (10), 125 (79), 124 (25), 123 (49), 111 (18), 107 (24), 97 (100), 95 (62), 77 (30), 75 (41), 51 (11).

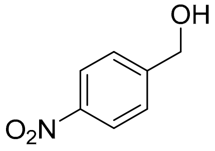

(4-nitrophenyl)methanol (1i) was obtained with 72% yield, and is known in the literature. All spectroscopic data are in agreement.[3] ^{1}H NMR (400 MHz, $CDCl_3$, 25°C) δ (ppm): 2.10 (br. s, 1H), 4.76 (s, 2H), 7.45 (d, J= 8.5 Hz, 2H), 8.12 (d, J= 8.7 Hz, 2H). ^{13}C NMR (101 MHz, $CDCl_3$, 25°C) δ (ppm): 64.0, 123.7, 127.0, 147.3, 148.2. EI-MS m/z (rel. int.): 153 (26), 136 (18), 107 (51), 89 (44), 77 (100), 63 (14), 51 (28).

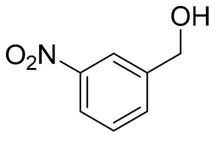

(3-nitrophenyl)methanol (1j) was obtained with 87% yield, and is known in the literature. All spectroscopic data are in agreement.[6] ^{1}H NMR (400 MHz, $CDCl_3$, 25°C) δ (ppm): 2.37 (br. s, 1H), 4.73 (s, 2H), 7.44 (t, J= 7.9 Hz, 1H), 7.61 (d, J= 7.7 Hz, 1H), 8.04 (d, J= 8.5 Hz, 1H), 8.14 (s, 1H). ^{13}C NMR (101 MHz, $CDCl_3$, 25°C) δ (ppm): 63.9, 121.5, 122.5, 129.5, 132.7, 142.9, 148.4. EI-MS m/z (rel. int.): 153 (26), 134 (30), 107 (46), 89 (50), 77 (100), 63 (13), 51 (25).

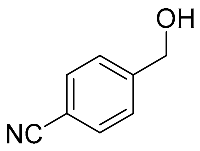

4-(hydroxymethyl)benzonitrile (1k) was obtained with 89% yield, and is known in the literature. All spectroscopic data are in agreement.[7] ^{1}H NMR (400 MHz, $CDCl_3$, 25°C) δ (ppm): 2.15 (br. s, 1H), 4.70 (s, 2H), 7.40 (d, J= 8.5 Hz, 2H), 7.56 (d, J= 8.5 Hz, 2H). ^{13}C NMR (101 MHz, $CDCl_3$,

25°C) δ (ppm): 64.2, 111.0, 118.9, 127.0, 132.3, 146.3. EI-MS m/z (rel. int.): 133 (32), 130 (13), 104 (100), 102 (15), 77 (31), 51 (15).

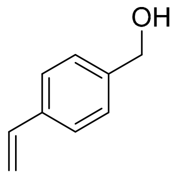

(4-vinylphenyl)methanol (1l) was obtained with 74% yield, and is known in the literature. All spectroscopic data are in agreement.[8] ^{1}H NMR (400 MHz, $CDCl_3$, 25°C) δ (ppm): 1.68 (br. s, 1H), 4.60 (s, 2H), 5.17 (dd, J= 10.9 Hz, 0.9 Hz, 1H), 5.68 (dd, J= 17.6 Hz, 0.9 Hz, 1H), 6.64 (dd, J= 17.6 Hz, 10.9 Hz, 1H), 7.24 (d, J= 8.1 Hz, 2H), 7.33 (d, J= 8.2 Hz, 2H). ^{13}C NMR (101 MHz, $CDCl_3$, 25°C) δ (ppm): 65.1, 113.9, 126.4, 127.2, 136.5, 137.1, 140.4. EI-MS m/z (rel. int.): 134 (100), 117 (18), 115 (26), 107 (15), 105 (95), 103 (41), 91 (31), 79 (46), 77 (59), 63 (13), 51 (22).

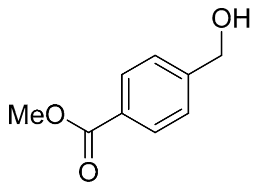

methyl 4-(hydroxymethyl)benzoate (1m) was obtained with 79% yield, and is known in the literature. All spectroscopic data are in agreement.[3] ^{1}H NMR (400 MHz, $CDCl_3$, 25°C) δ (ppm): 1.66 (br. s, 1H), 3.85 (s, 3H), 4.70 (s, 2H), 7.37 (d, J= 8.6 Hz, 2H), 7.96 (d, J= 8.3 Hz, 2H). ^{13}C NMR (101 MHz, $CDCl_3$, 25°C) δ (ppm): 52.1, 64.7, 126.5, 129.4, 129.9, 145.9, 167.0. EI-MS m/z (rel. int.): 166 (40), 137 (49), 135 (100), 133 (40), 107 (95), 105 (38), 91 (13), 89 (46), 79 (49), 77 (81), 63 (10), 51 (25).

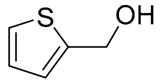

thiophen-2-ylmethanol (1n) was obtained with 86% yield, and is known in the literature. All spectroscopic data are in agreement.[3] ^{1}H NMR (400 MHz, $CDCl_3$, 25°C) δ (ppm): 2.83 (br. s, 1H), 4.78 (s, 2H), 7.00 (dd, J= 6.8 Hz, 3.3 Hz, 2H), 7.29 (t, J= 4.4 Hz, 1H). ^{13}C NMR (101 MHz, $CDCl_3$, 25°C) δ (ppm): 59.8, 125.5, 125.5, 126.9, 144.0. EI-MS m/z (rel. int.): 114 (100), 97 (76), 85 (89), 81 (36), 69 (12), 58 (14), 57 (12), 53 (14).

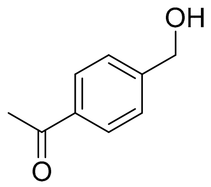

1-(4-(hydroxymethyl)phenyl)ethan-1-one (1o) was obtained with 72% yield, and is known in the literature. All spectroscopic data are in agreement.[6] ^{1}H NMR (400 MHz, $CDCl_3$, 25°C) δ (ppm): 2.50 (s, 3H), 2.61 (br. s, 1H), 4.67 (s, 2H), 7.35 (d, J= 8.5 Hz, 2H), 7.83 (d, J = 8.3 Hz, 2H). ^{13}C NMR (101 MHz, $CDCl_3$, 25°C) δ (ppm): 26.6, 64.4, 126.6, 128.6, 136.2, 146.5, 198.2. EI-MS m/z (rel. int.): 150 (19), 135 (100), 107 (16), 89 (31), 77 (31), 51 (11).

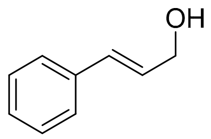

(E)-3-phenylprop-2-en-1-ol (1p) was obtained with 61% yield, and is known in the literature. All spectroscopic data are in agreement.[3] ^{1}H NMR (400 MHz, $CDCl_3$, 25°C) δ (ppm): 1.64 (br. s, 1H), 4.24 (dd, J= 5.7Hz, 1.5 Hz, 2H), 6.29 (dt, J= 15.9 Hz, 5.7 Hz, 1H), 6.54 (dt, J= 15.8 Hz, 1.6 Hz, 1H), 7.09–7.24 (m, 1H), 7.21–7.26 (m, 2H), 7.27-7.36 (m, 2H). ^{13}C NMR (101 MHz, $CDCl_3$, 25°C) δ (ppm): 63.7, 126.5, 127.7, 128.5, 128.6, 131.2, 136.7. EI-MS m/z (rel. int.): 134 (69), 117 (16), 115 (68), 105 (55), 103 (41), 92 (100), 91 (80), 78 (60), 77 (54), 65 (14), 63 (18), 57 (45), 51 (32).

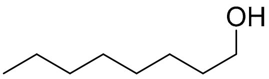

octan-1-ol (1r) was obtained with 71% yield, and is known in the literature. All spectroscopic data are in agreement.[3] ^{1}H NMR (400 MHz, $CDCl_3$, 25°C) δ (ppm): 0.82 (t, J= 6.8 Hz, 3H), 1.19-1.31 (m, 10H), 1.46-1.52 (m, 2H), 1.55 (br. s, 1H), 3.57 (t, J= 6.6 Hz, 2H). ^{13}C NMR (101 MHz, $CDCl_3$, 25°C) δ (ppm): 14.1, 22.7, 25.7, 29.3, 29.4, 31.8, 32.8, 63.1. EI-MS m/z (rel. int.): 112 (3), 97 (5), 84 (56), 83 (48), 70 (71), 69 (73), 56 (100), 55 (95).

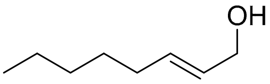

(E)-oct-2-en-1-ol (1s) was obtained with 77% yield, and is known in the literature. All spectroscopic data are in agreement.[9] ^{1}H NMR (400 MHz, $CDCl_3$, 25°C) δ (ppm): 0.82 (t, J = 6.8 Hz, 3H), 1.20-1.25 (m, 4H), 1.28-1.35 (m, 2H), 1.59 (br. s, 1H), 1.97 (q, J= 6.7 Hz, 2H), 4.01 (d, J= 4.9 Hz, 2H), 5.50-5.69 (m, 2H). ^{13}C NMR (101 MHz, $CDCl_3$, 25°C) δ (ppm): 14.0, 22.5, 28.8, 31.4, 32.2, 63.8, 128.8, 133.6. EI-MS m/z (rel. int.): 110 (17), 95 (14), 82 (23), 81 (34), 68 (32), 67 (42), 57 (100), 55 (44), 54 (37).

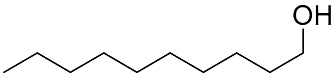

decan-1-ol (1t) was obtained with 83% yield, and is known in the literature. All spectroscopic data are in agreement.[10] ^{1}H NMR (400 MHz, $CDCl_3$, 25°C) δ (ppm): 0.80 (t, J= 7.0 Hz, 3H), 1.17-1.32 (m, 14H), 1.44-1.54 (m, 2H), 1.75 (br. s, 1H), 3.57 (t, J= 6.6 Hz, 2H). ^{13}C NMR (101 MHz, $CDCl_3$, 25°C) δ (ppm): 14.1, 22.7, 25.7, 29.3, 29.4, 29.6, 29.6, 31.9, 32.8, 63.1. EI-MS m/z (rel. int.): 140 (2), 112 (17), 111 (15), 98 (12), 97 (32), 84 (39), 83 (62), 70 (85), 69 (74), 56 (78), 55 (100).

5. Spectra for all products

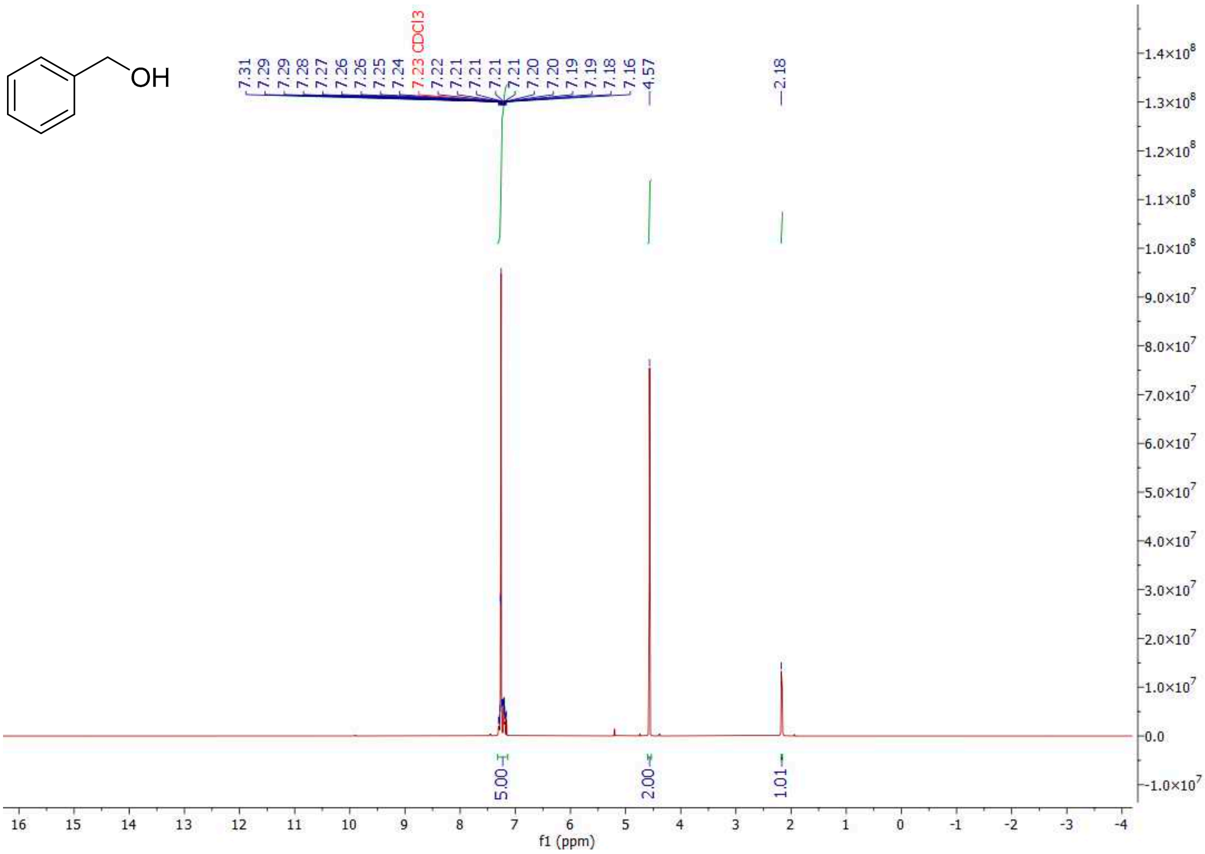

Figure S1. ^{1}H NMR Spectra of phenylmethanol (1a).

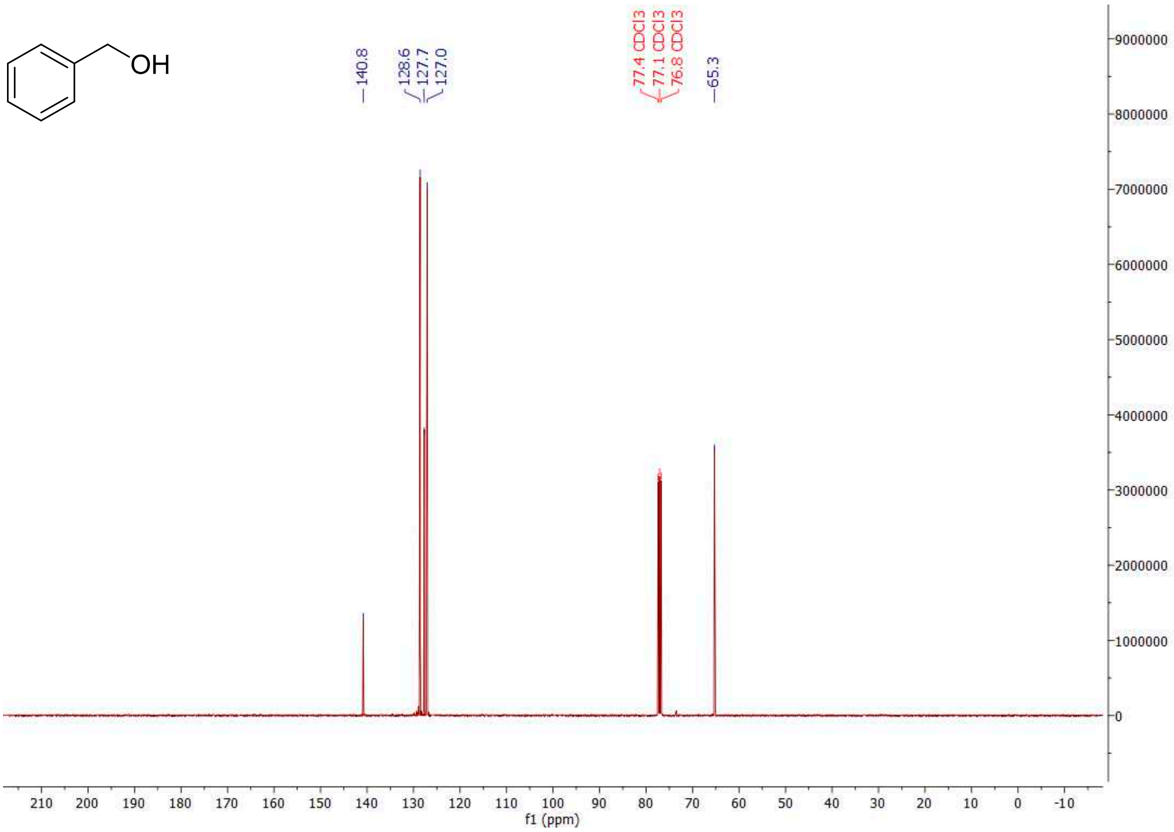

Figure S2. ^{13}C NMR Spectra of phenylmethanol (1a).

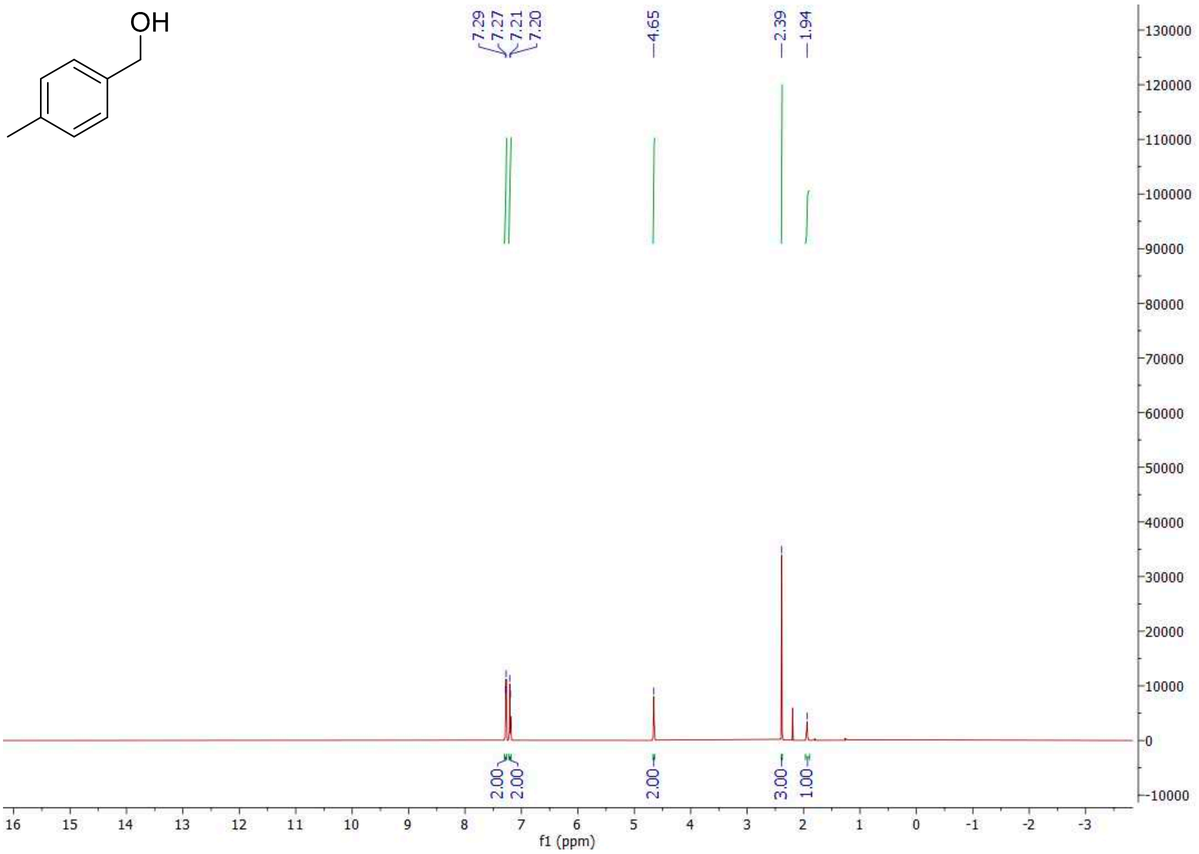

Figure S3. ^{1}H NMR Spectra of p-tolylmethanol (1b).

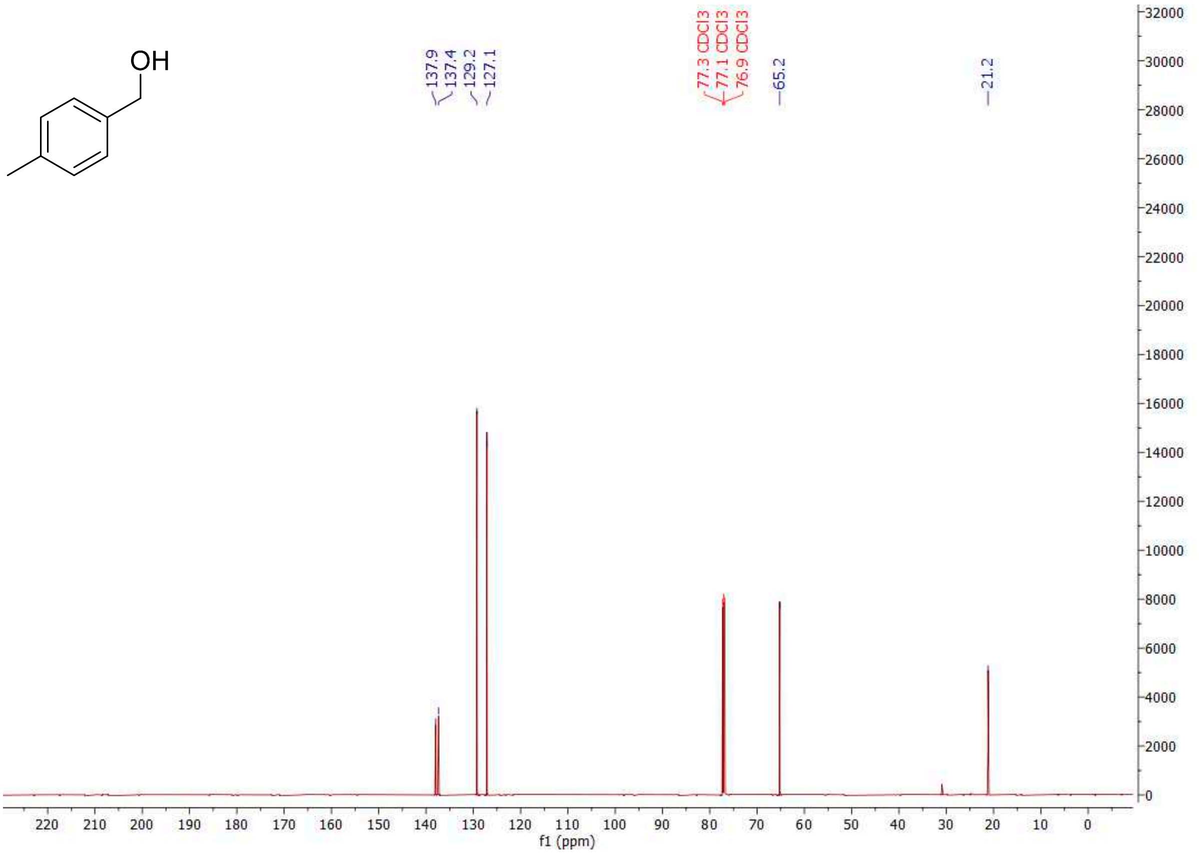

Figure S4. ^{13}C NMR Spectra of p-tolylmethanol (1b).

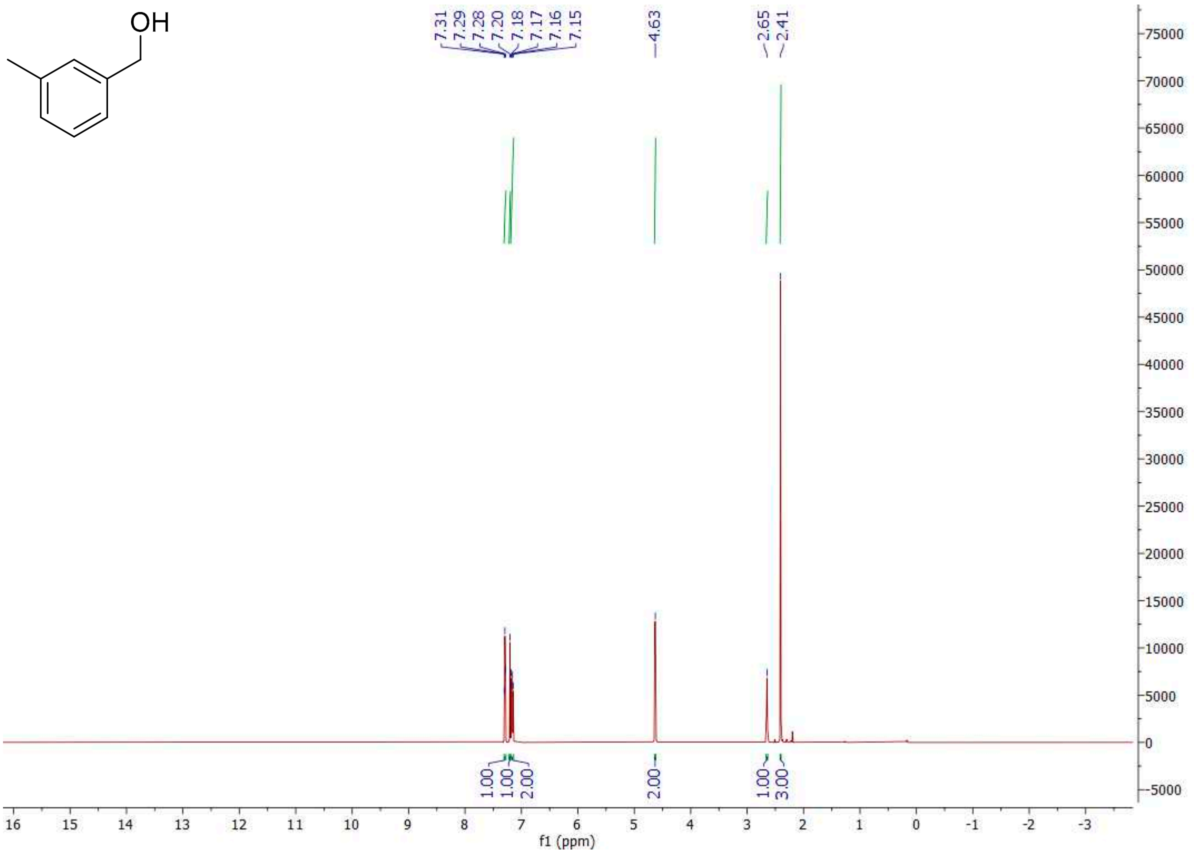

Figure S5. ^{1}H NMR Spectra of m-tolylmethanol (1c).

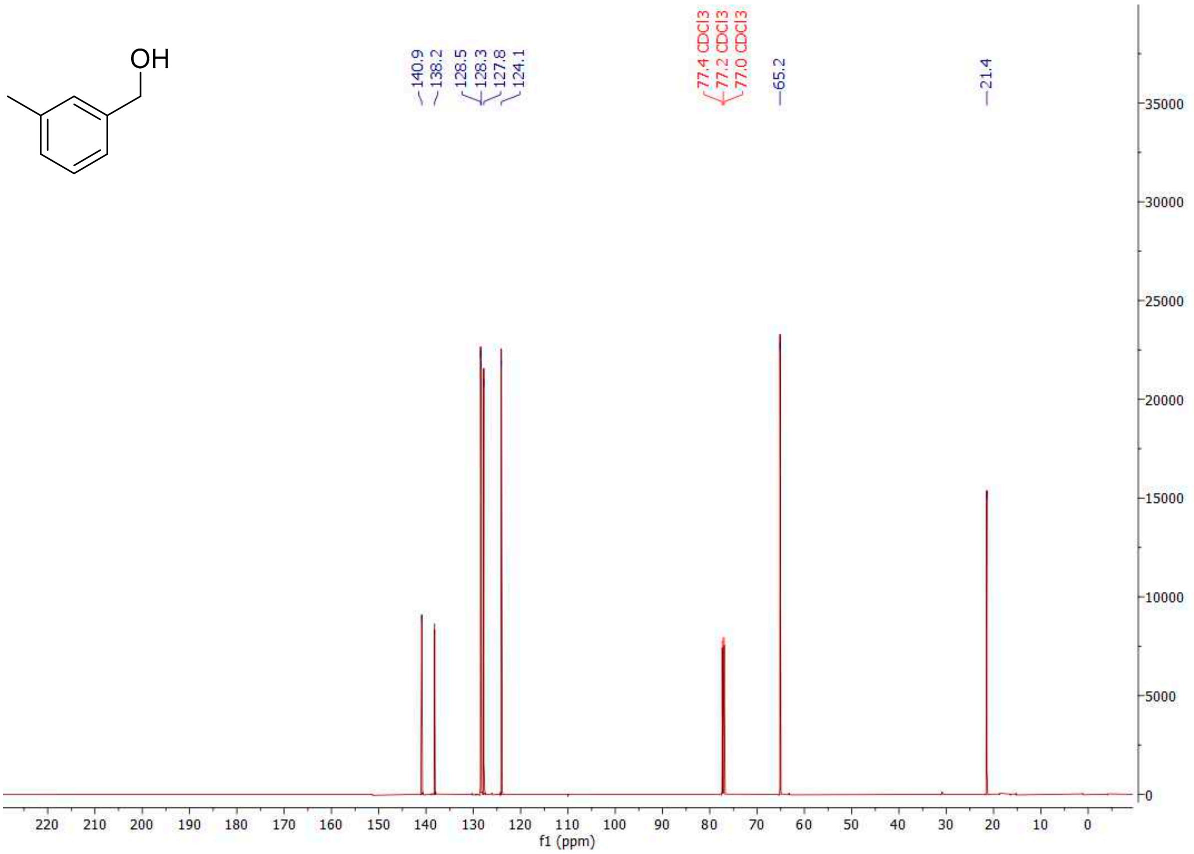

Figure S6. ^{13}C NMR Spectra of m-tolylmethanol (1c).

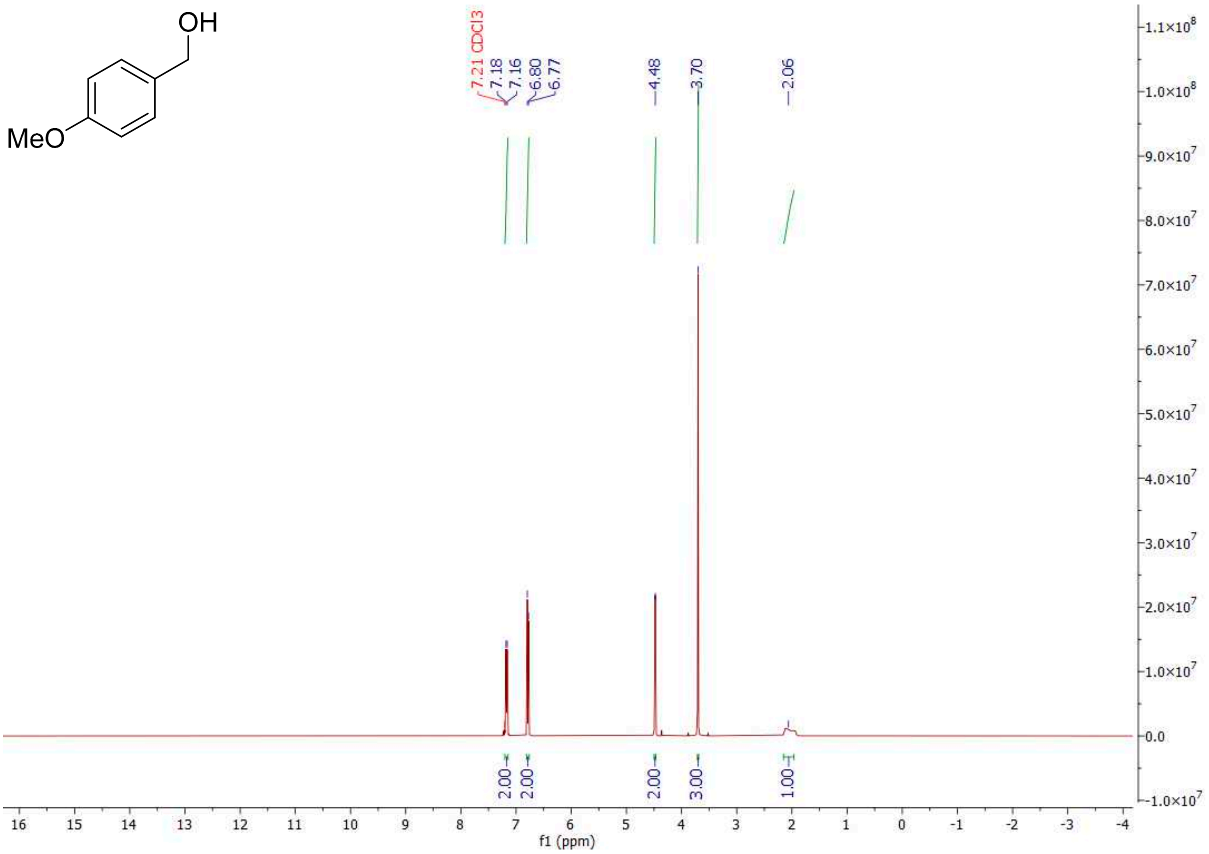

Figure S7. ^{1}H NMR Spectra of (4-methoxyphenyl)methanol (1d).

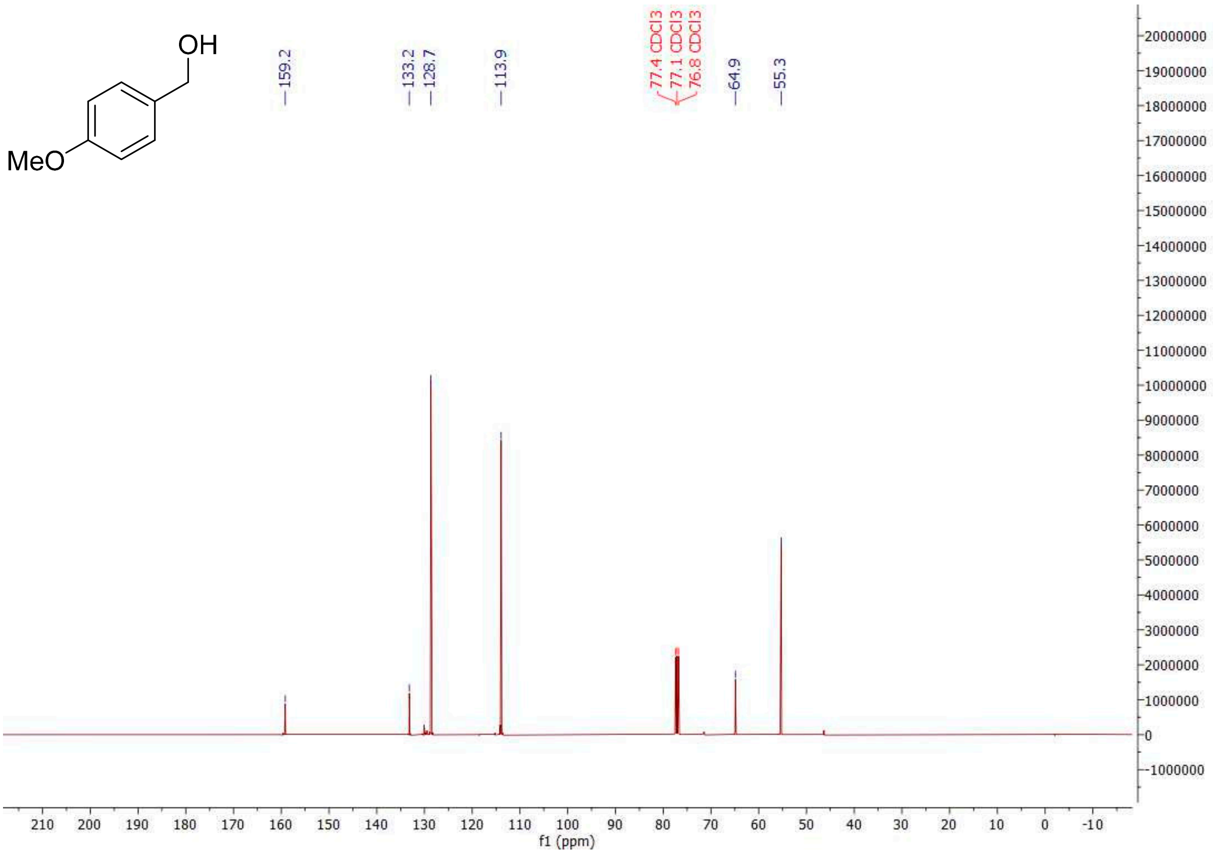

Figure S8. ^{13}C NMR Spectra of (4-methoxyphenyl)methanol (1d).

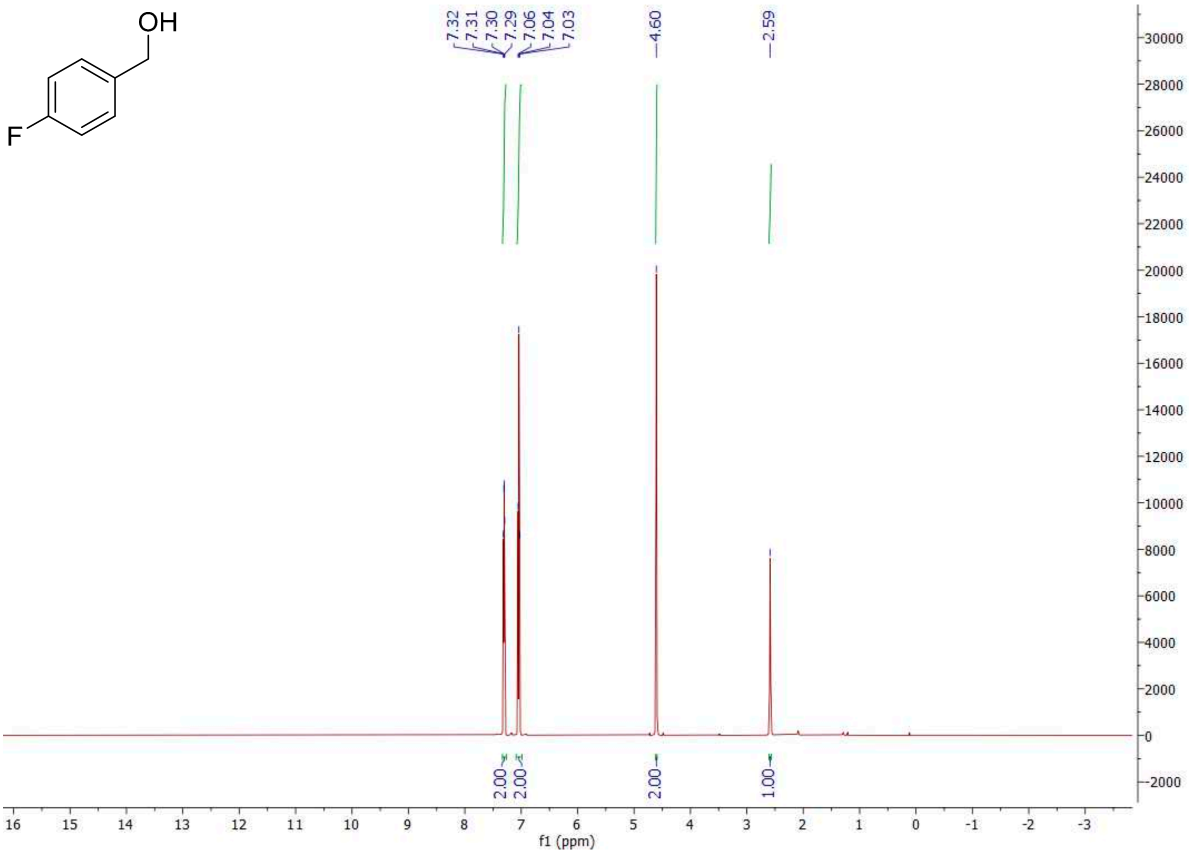

Figure S9. ^{1}H NMR Spectra of (4-fluorophenyl)methanol (1e).

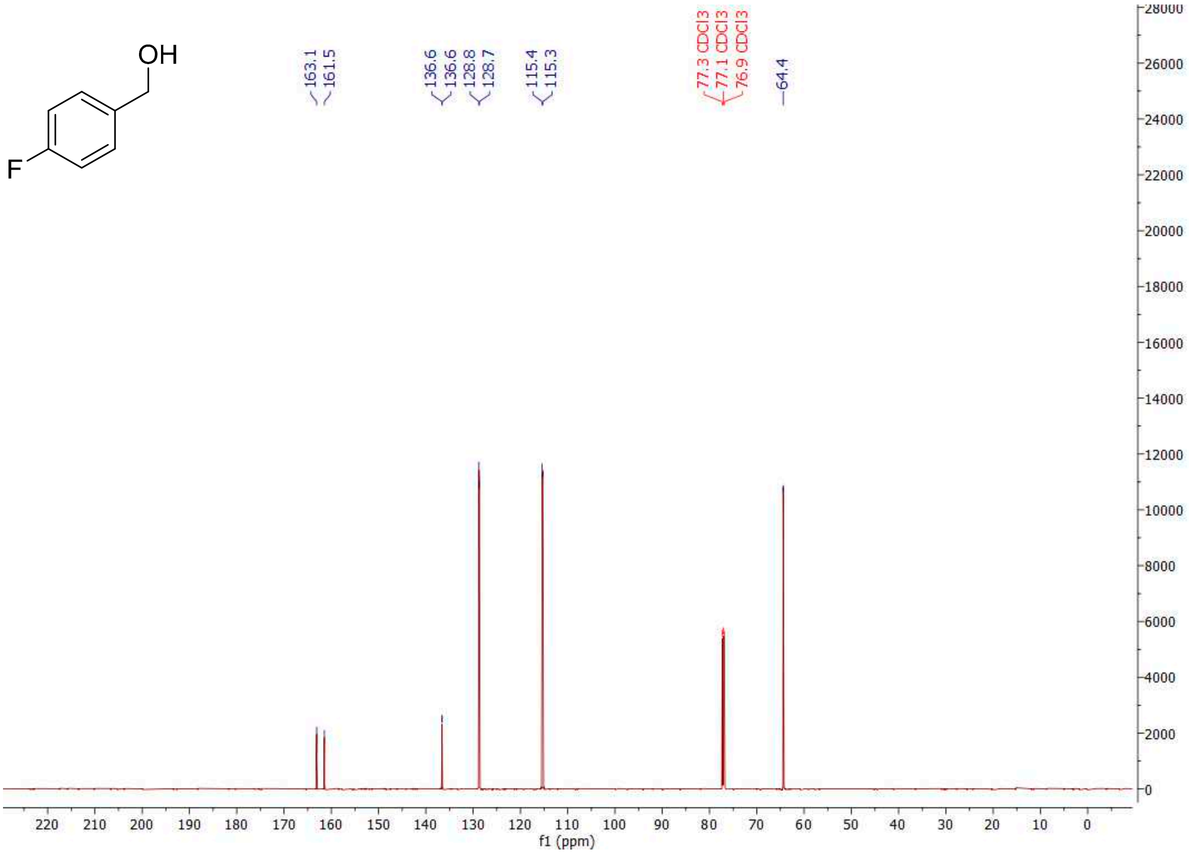

Figure S10. ^{13}C NMR Spectra of (4-fluorophenyl)methanol (1e).

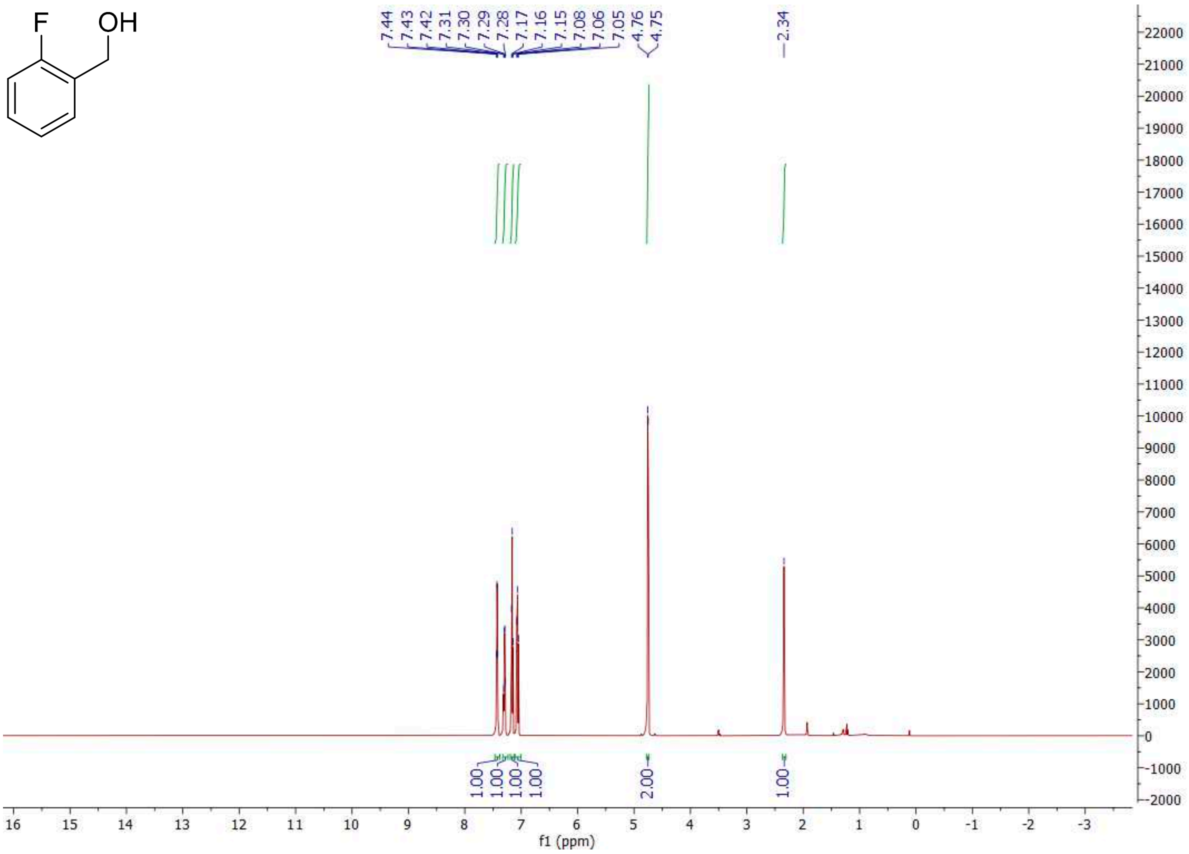

Figure S11. ^{1}H NMR Spectra of (2-fluorophenyl)methanol (1f).

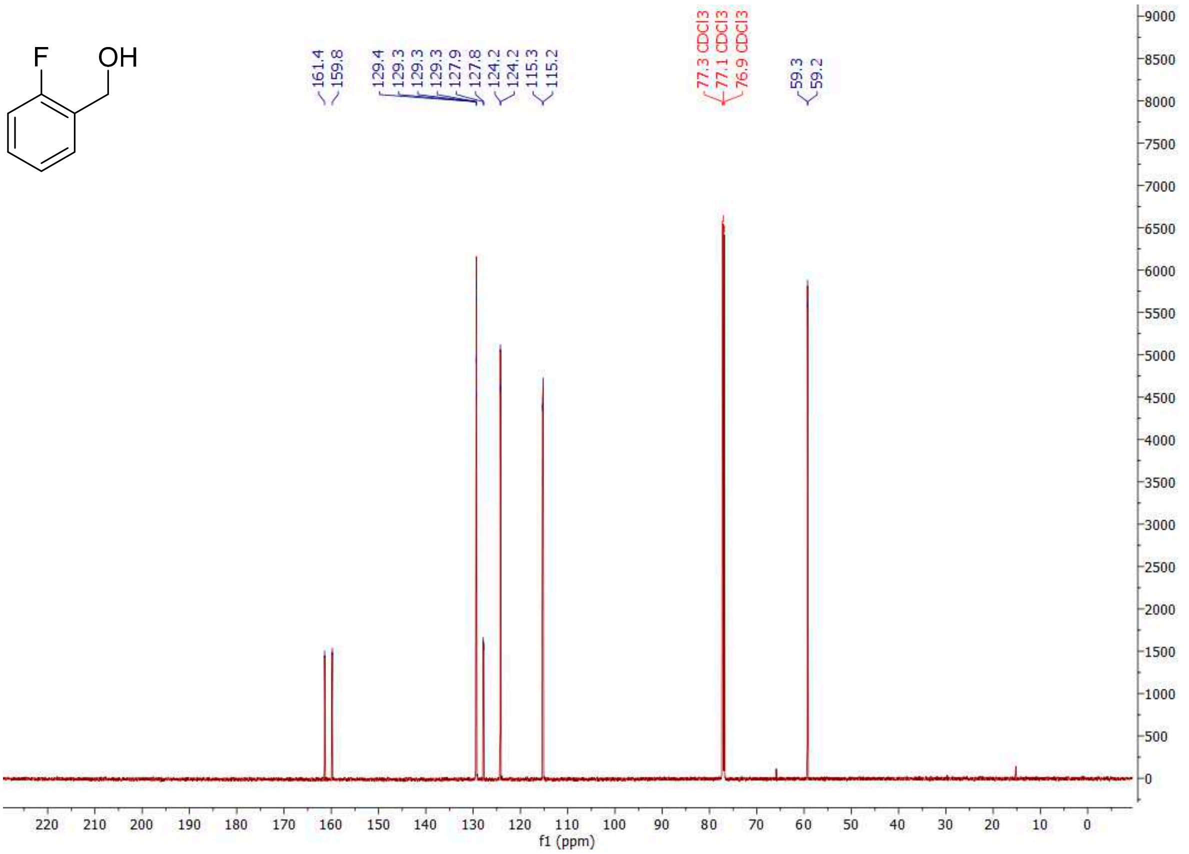

Figure S12. ^{13}C NMR Spectra of (2-fluorophenyl)methanol (1f).

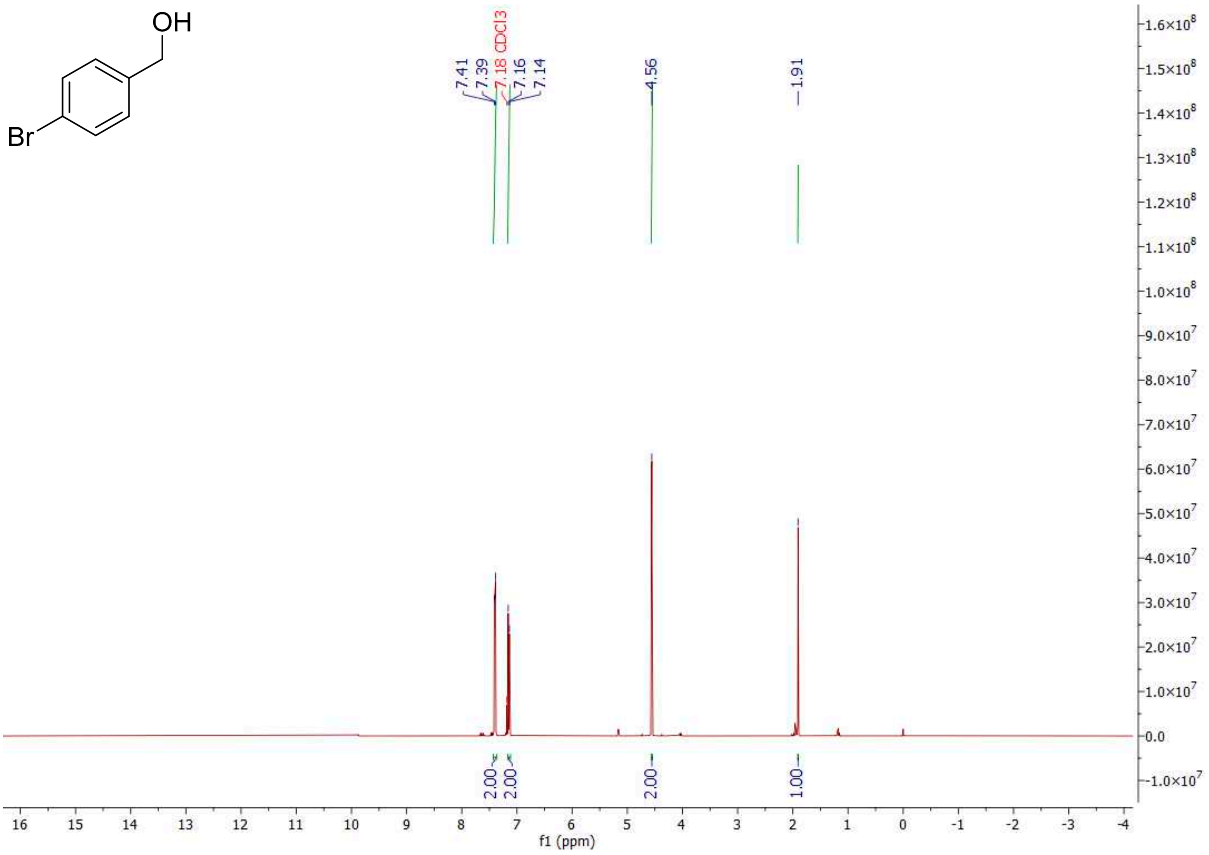

Figure S13. ^{1}H NMR Spectra of (4-bromophenyl)methanol (1g).

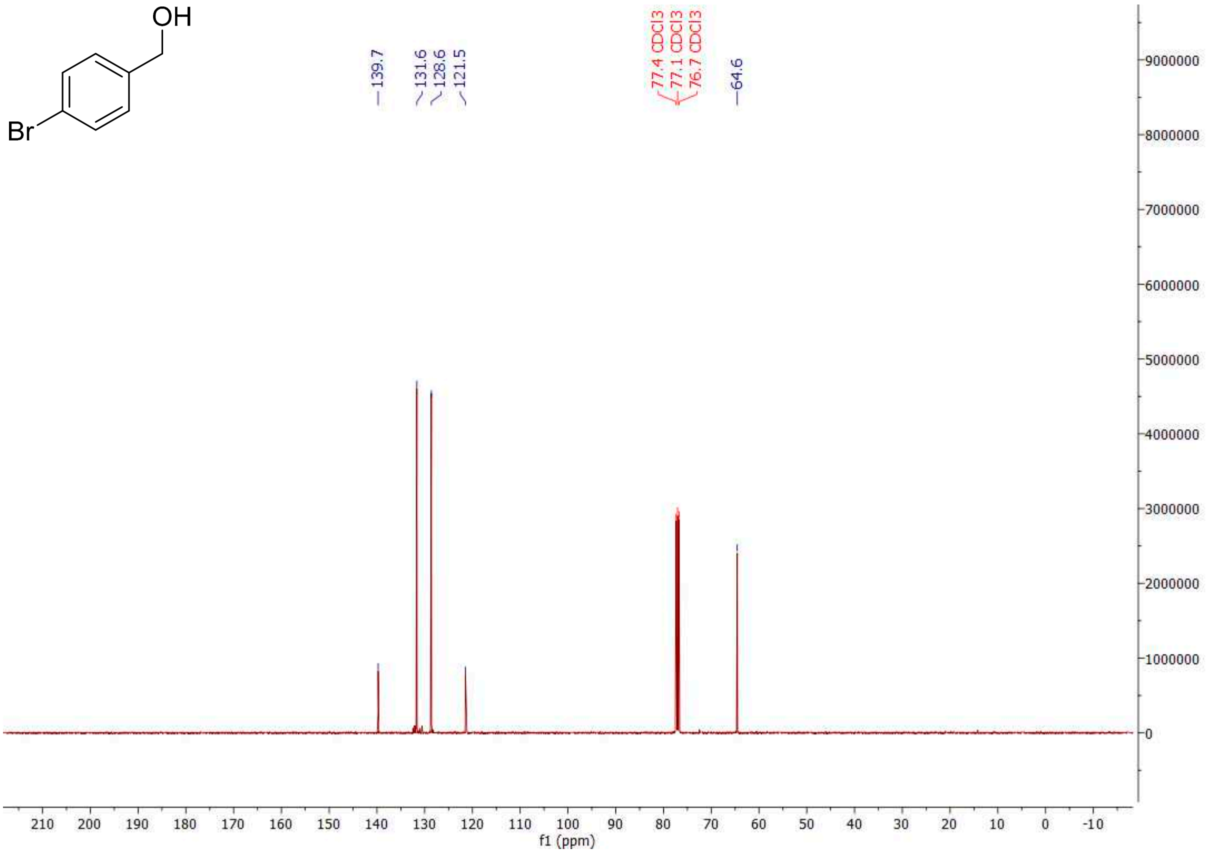

Figure S14. ^{13}C NMR Spectra of (4-bromophenyl)methanol (1g).

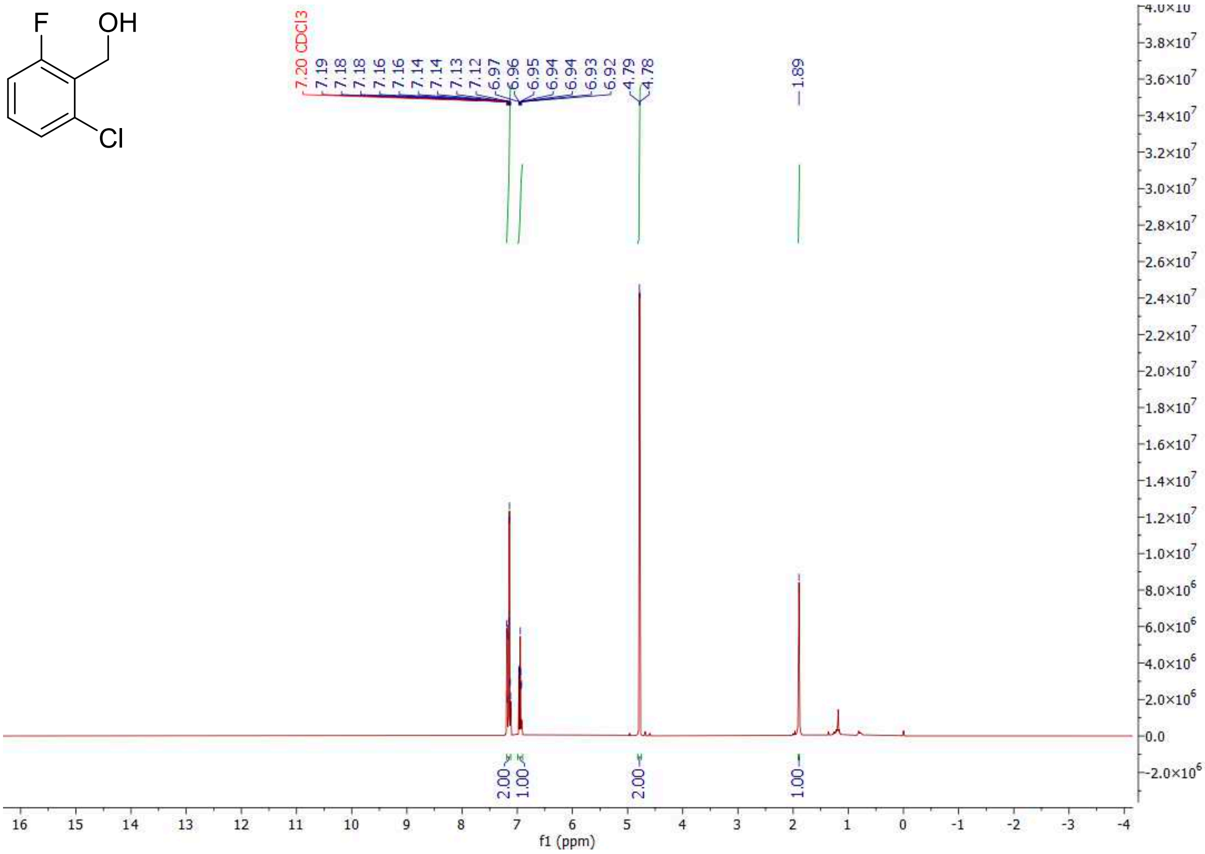

Figure S15. ^{1}H NMR Spectra of (2-chloro-6-fluorophenyl)methanol (1h).

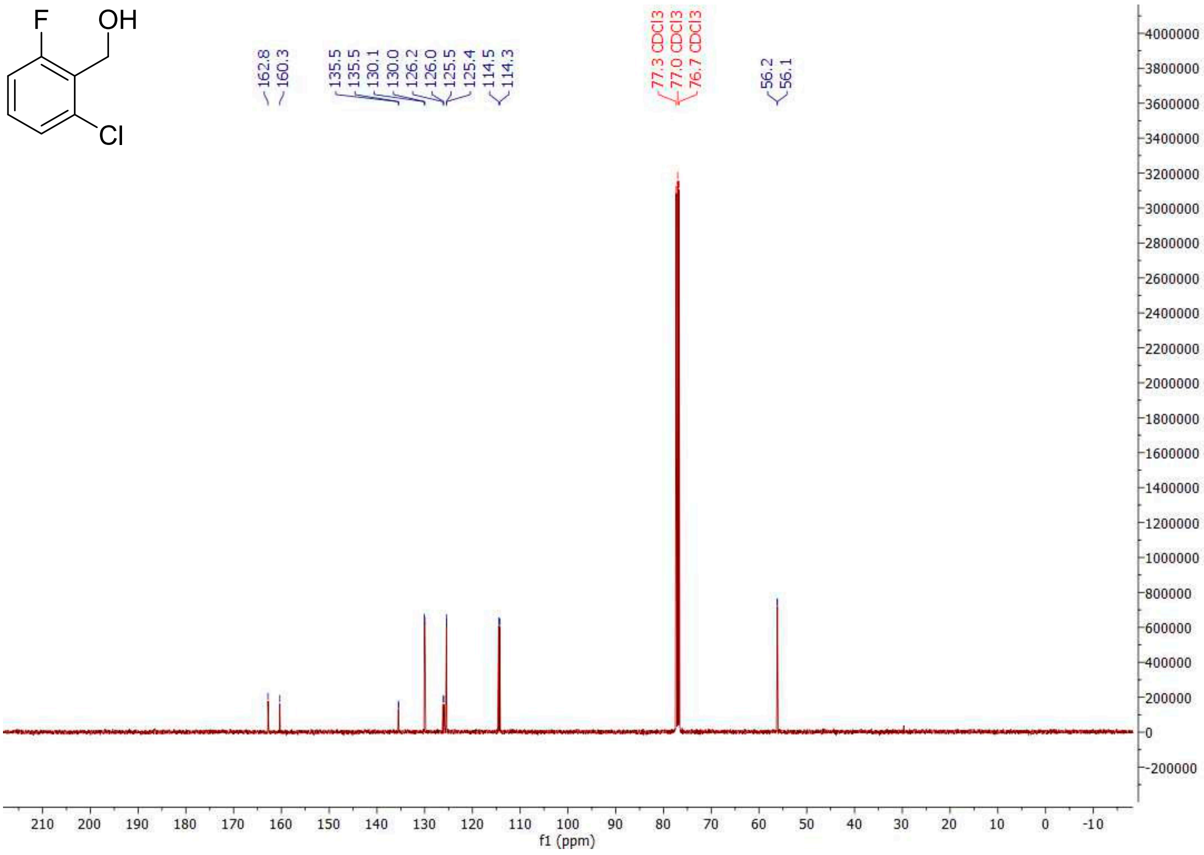

Figure S16. ^{13}C NMR Spectra of (2-chloro-6-fluorophenyl)methanol (1h).

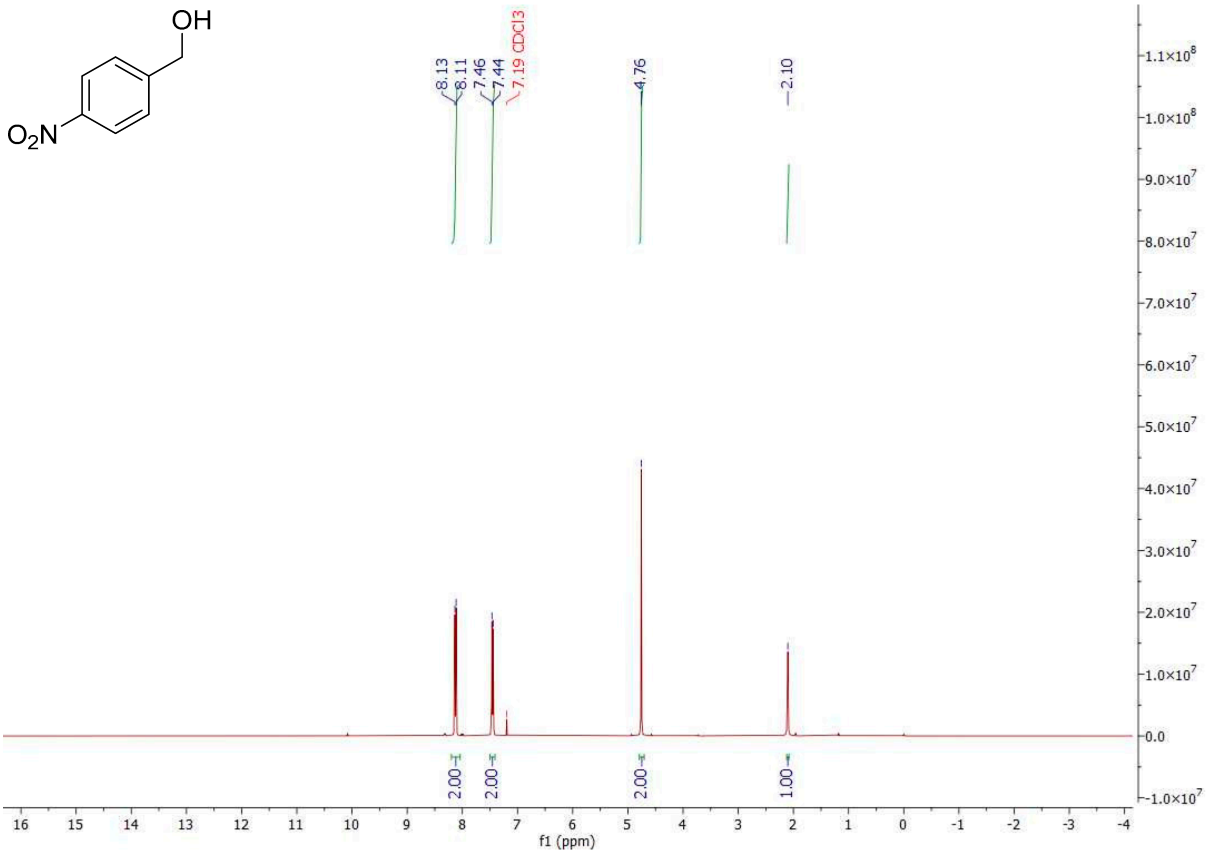

Figure S17. ^{1}H NMR Spectra of (4-nitrophenyl)methanol (1i).

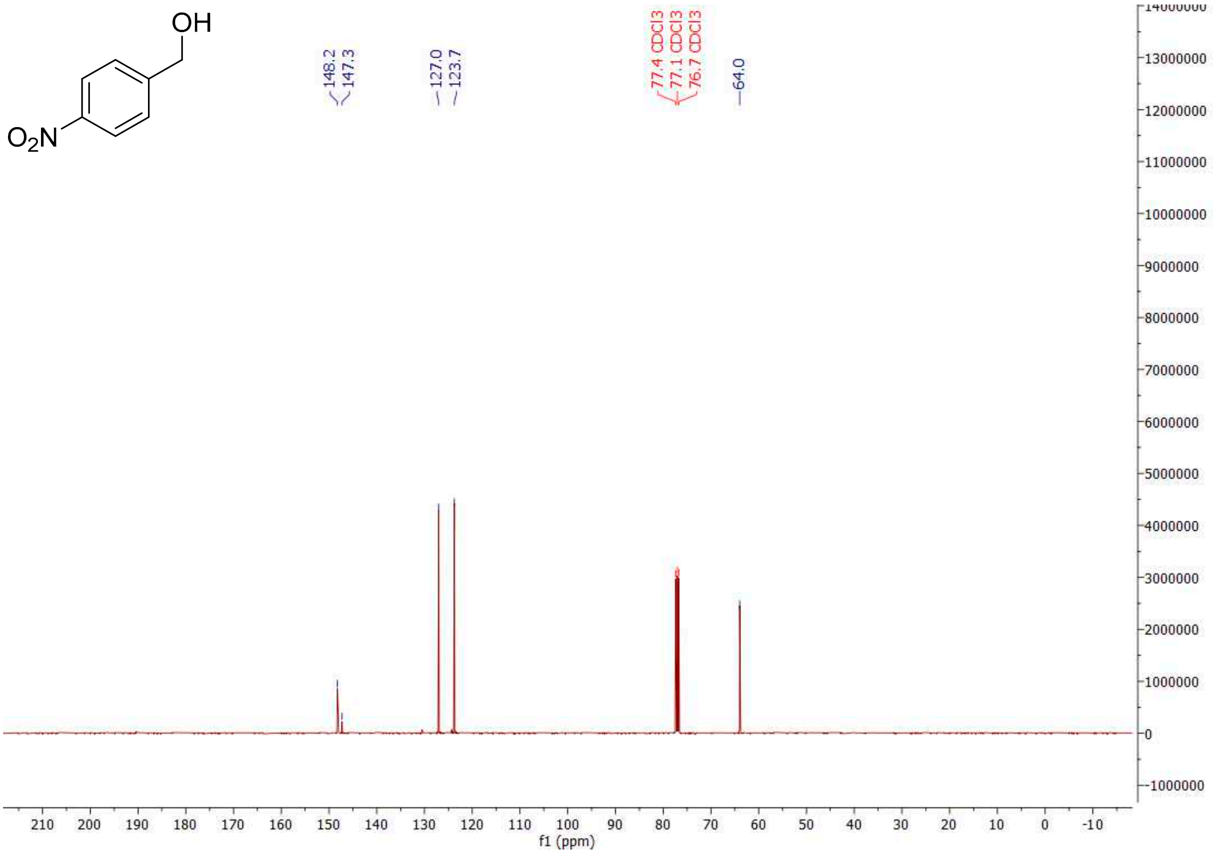

Figure S18. ^{13}C NMR Spectra of (4-nitrophenyl)methanol (1i).

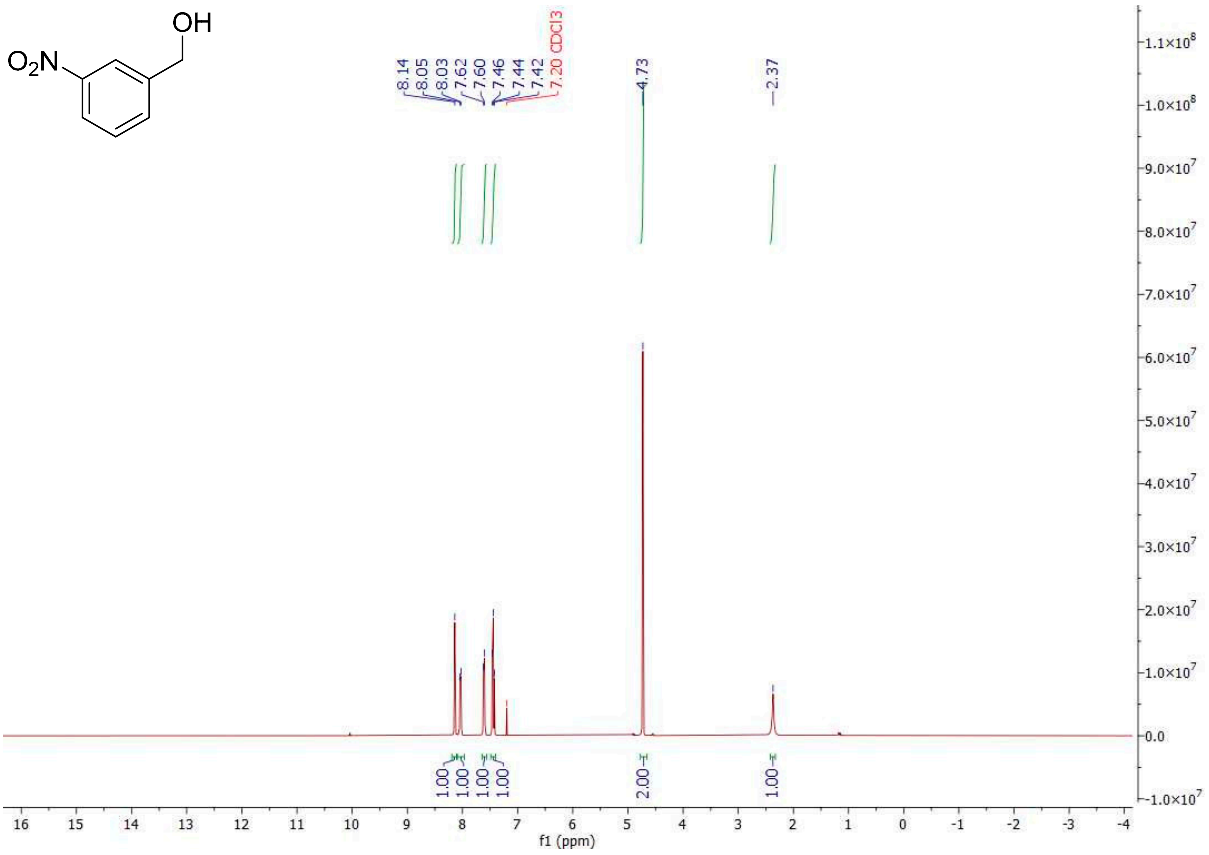

Figure S19. ^{1}H NMR Spectra of (3-nitrophenyl)methanol (1j).

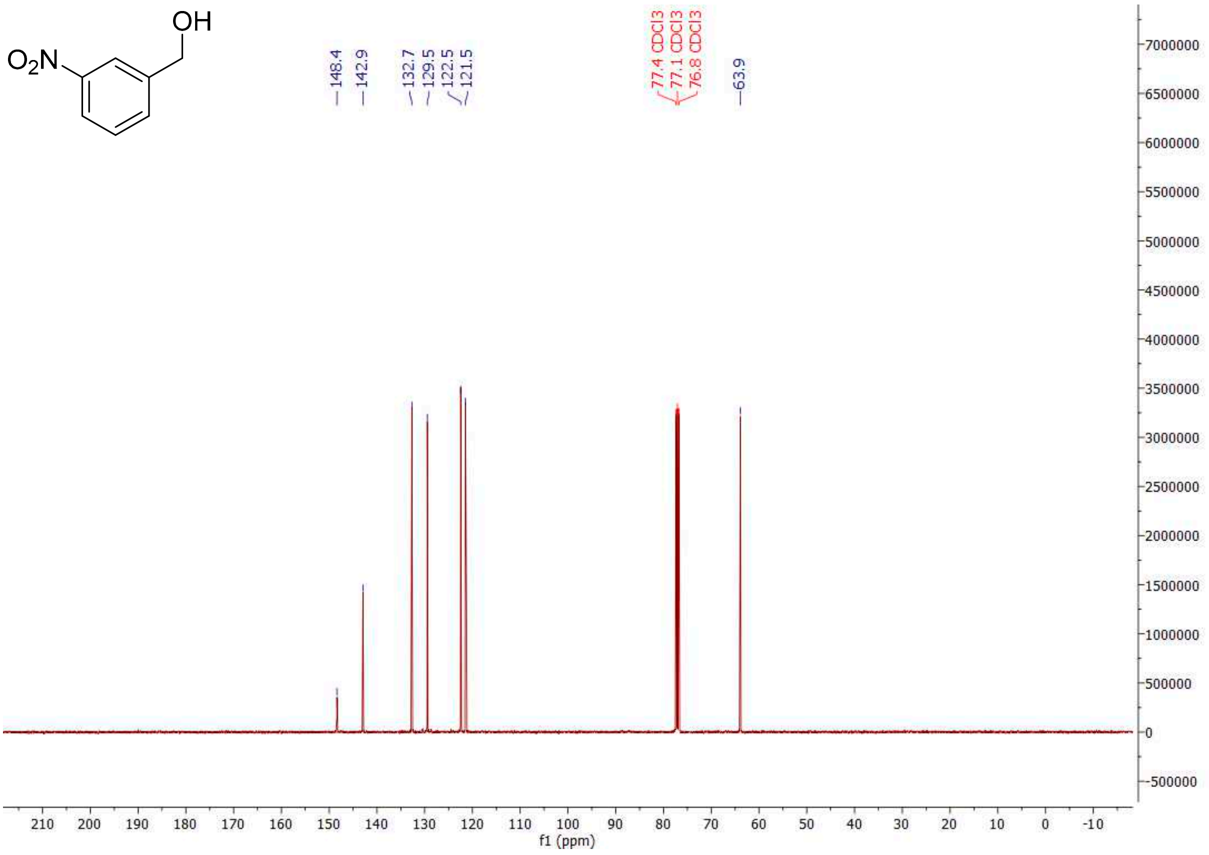

Figure S20. ^{13}C NMR Spectra of (3-nitrophenyl)methanol (1i).

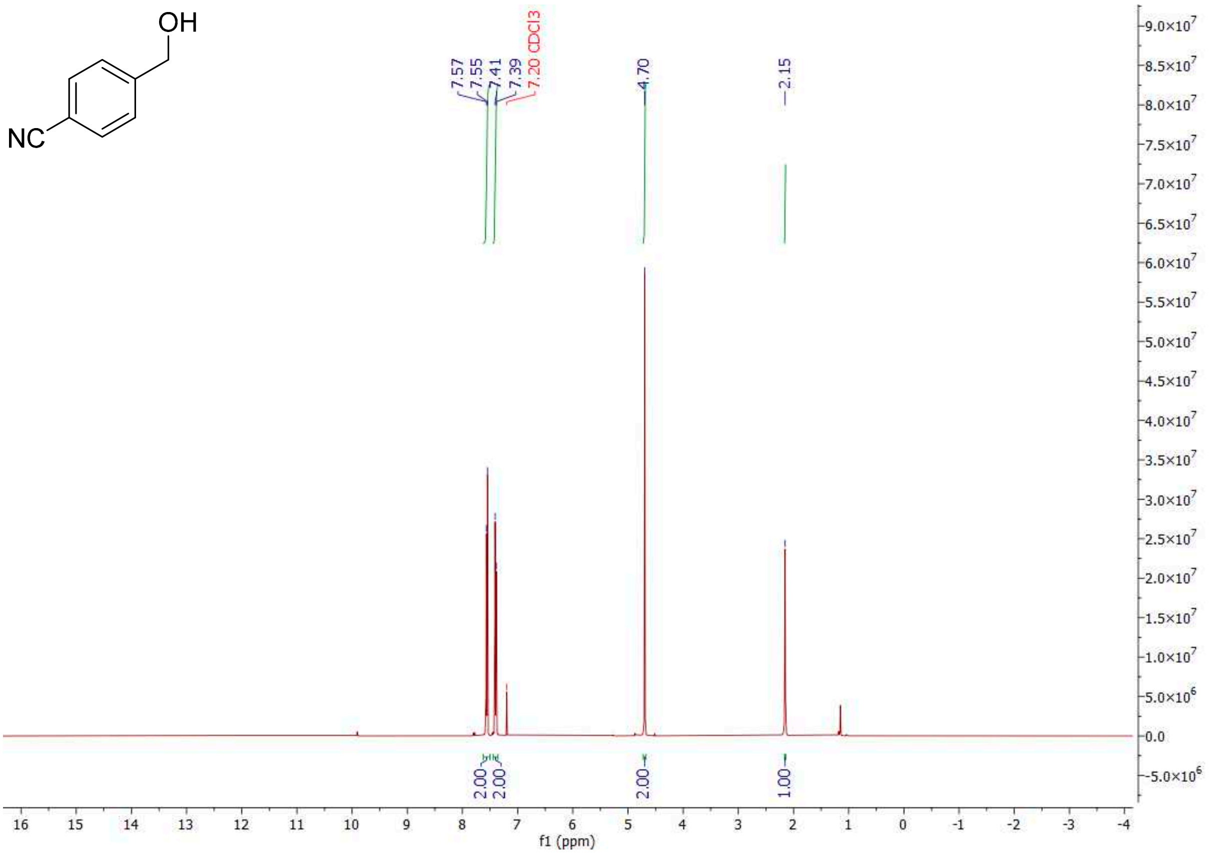

Figure S21. ^{1}H NMR Spectra of 4-(hydroxymethyl)benzonitrile (1k).

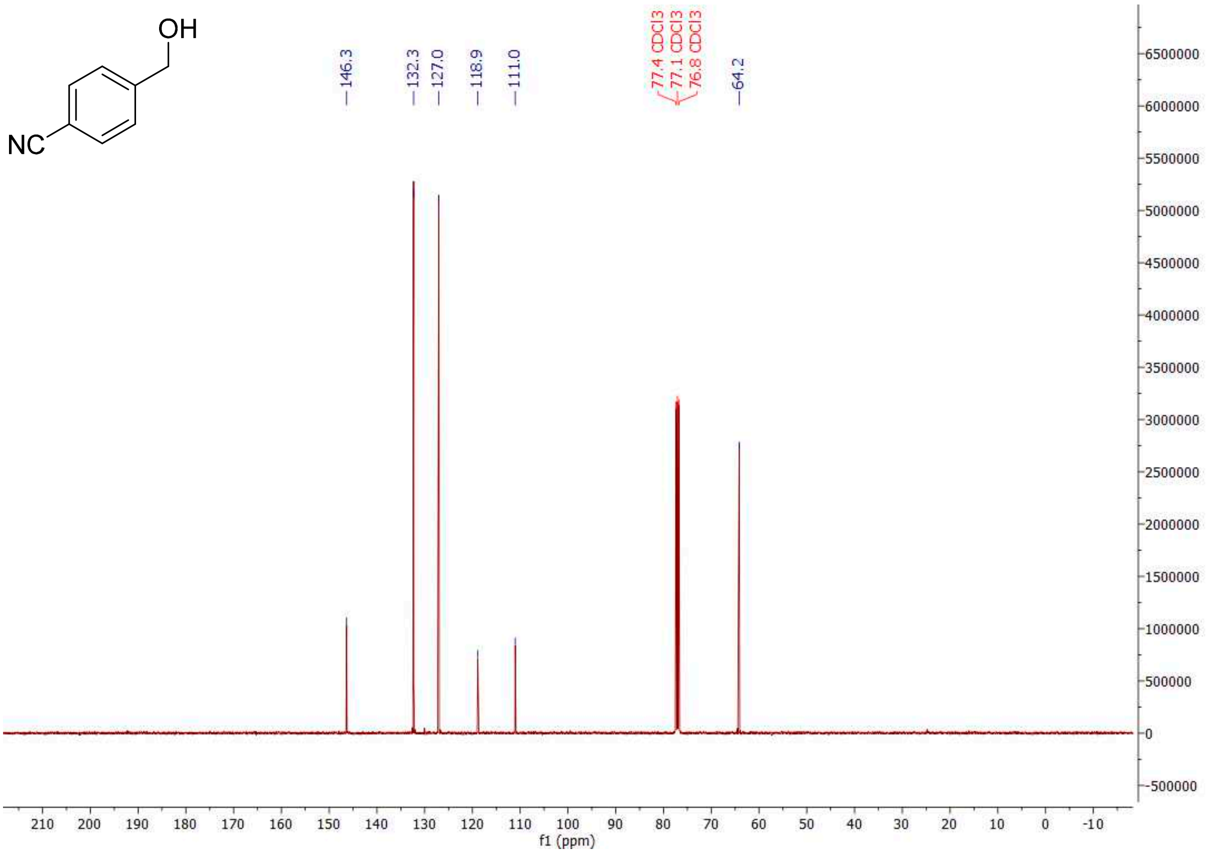

Figure S22. ^{13}C NMR Spectra of 4-(hydroxymethyl)benzonitrile (1k).

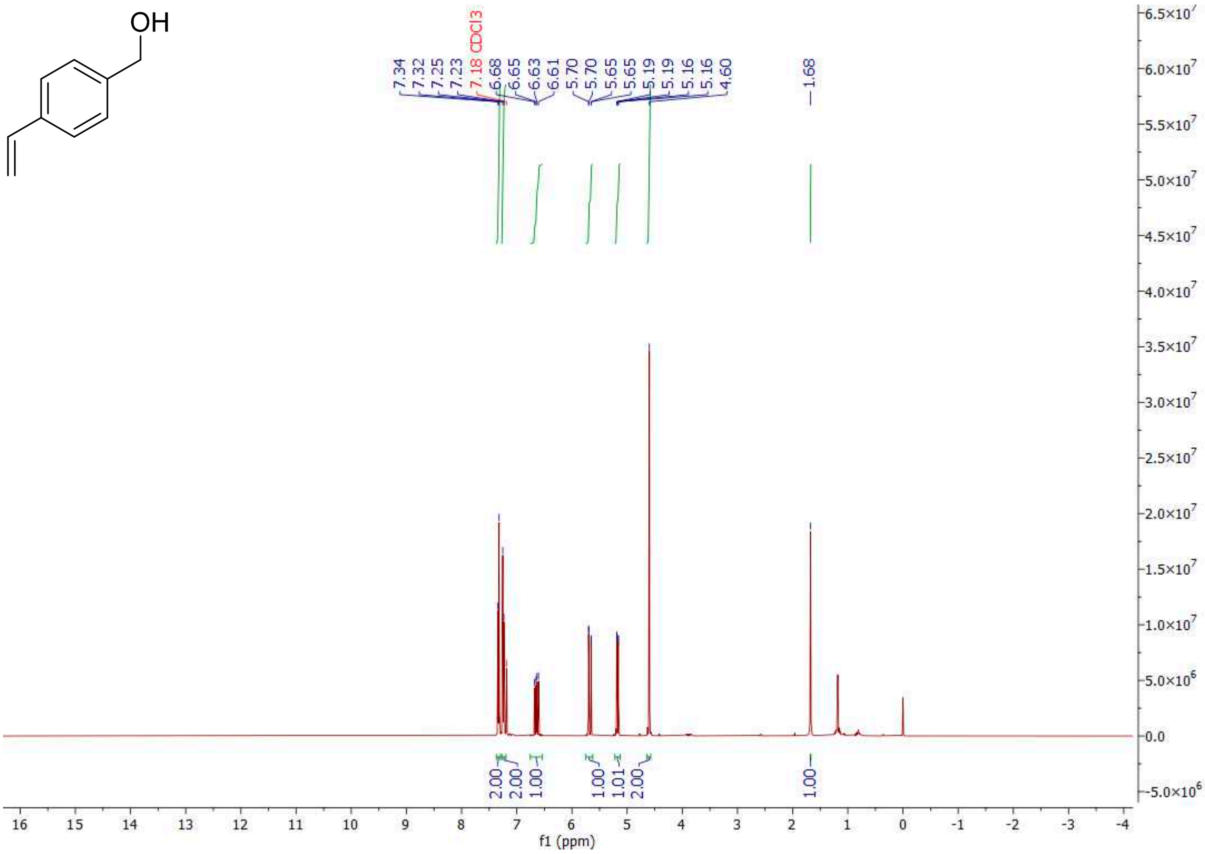

Figure S23. ^{1}H NMR Spectra of (4-vinylphenyl)methanol (1l).

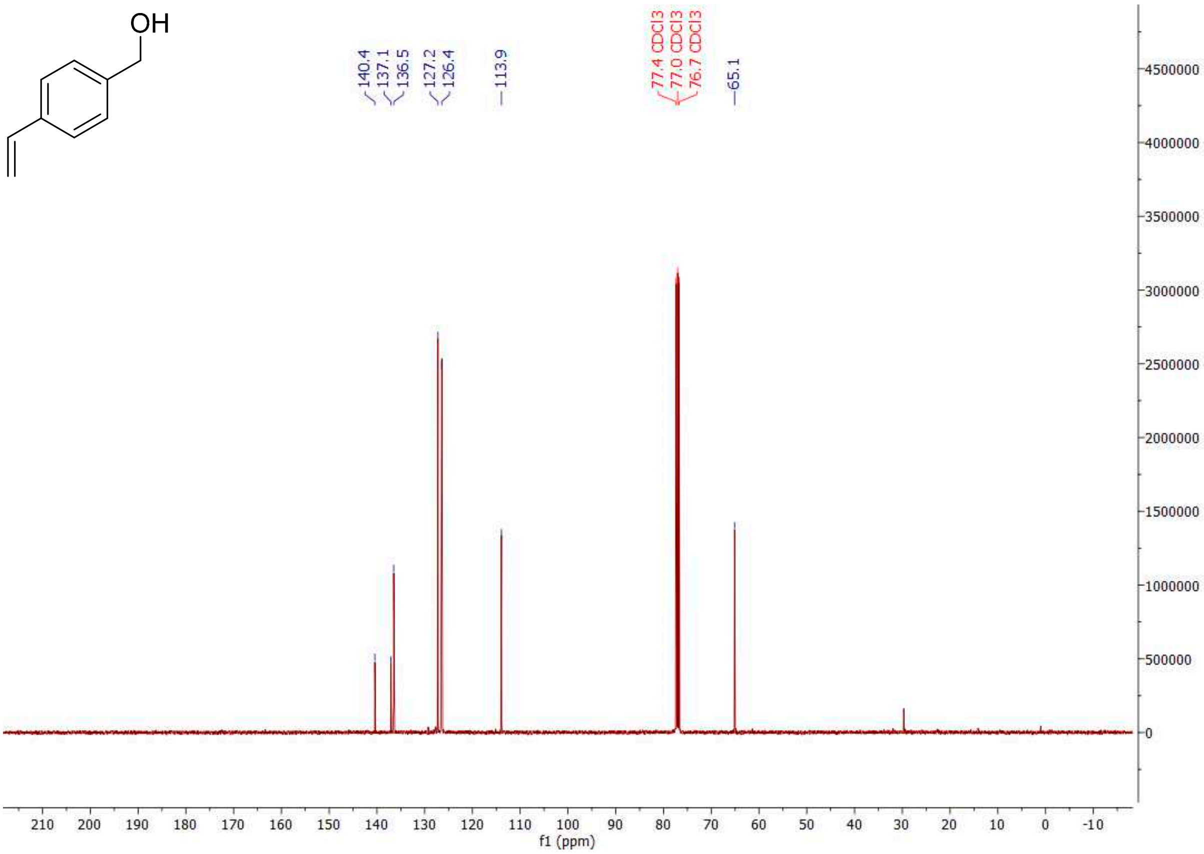

Figure S24. ^{13}C NMR Spectra of (4-vinylphenyl)methanol (1l).

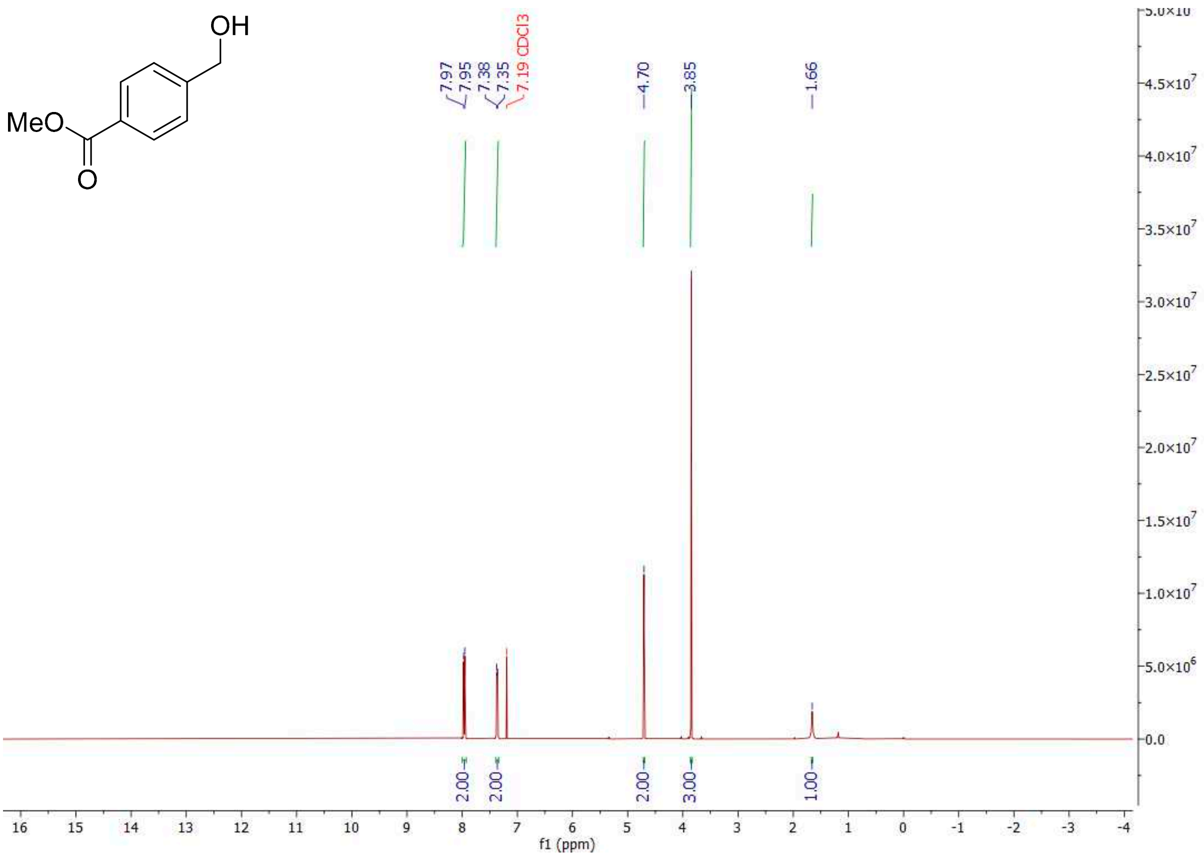

Figure S25. ^{1}H NMR Spectra of methyl 4-(hydroxymethyl)benzoate (1m).

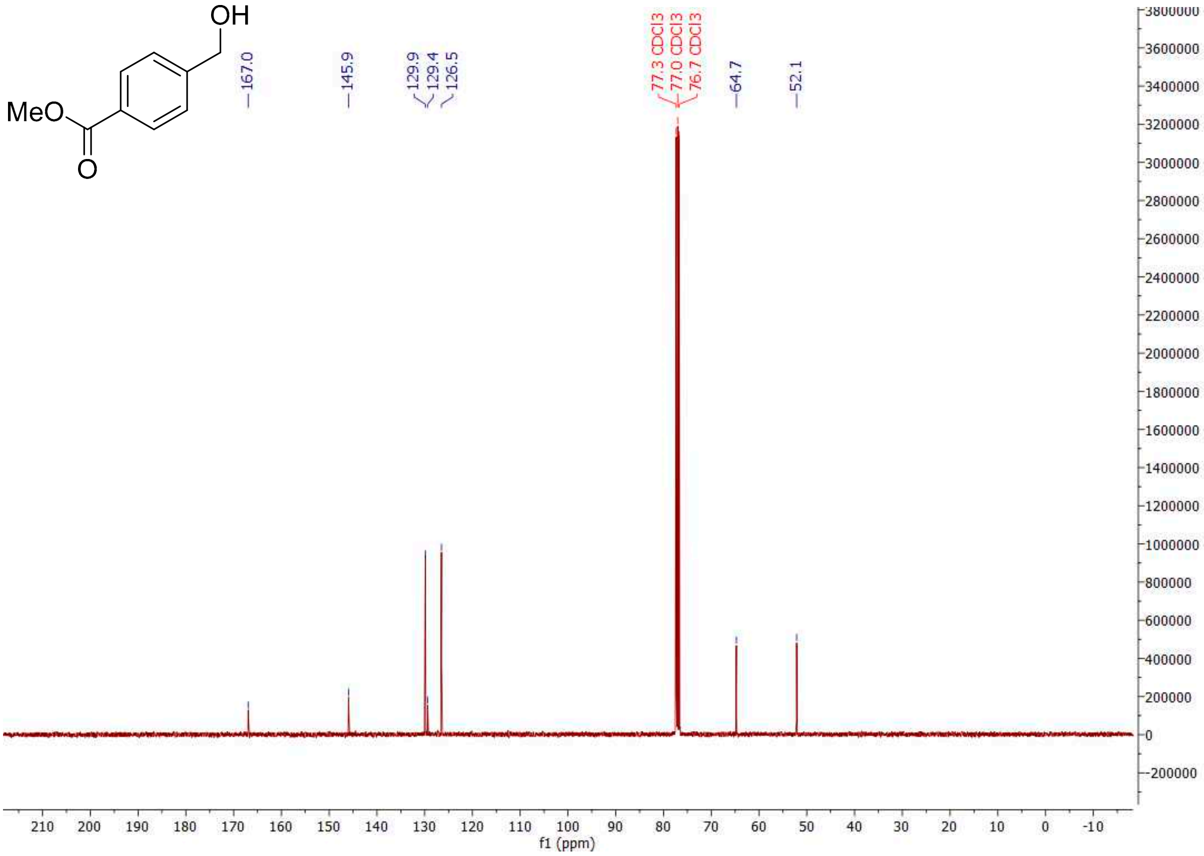

Figure S26. ^{13}C NMR Spectra of methyl 4-(hydroxymethyl)benzoate (1m).

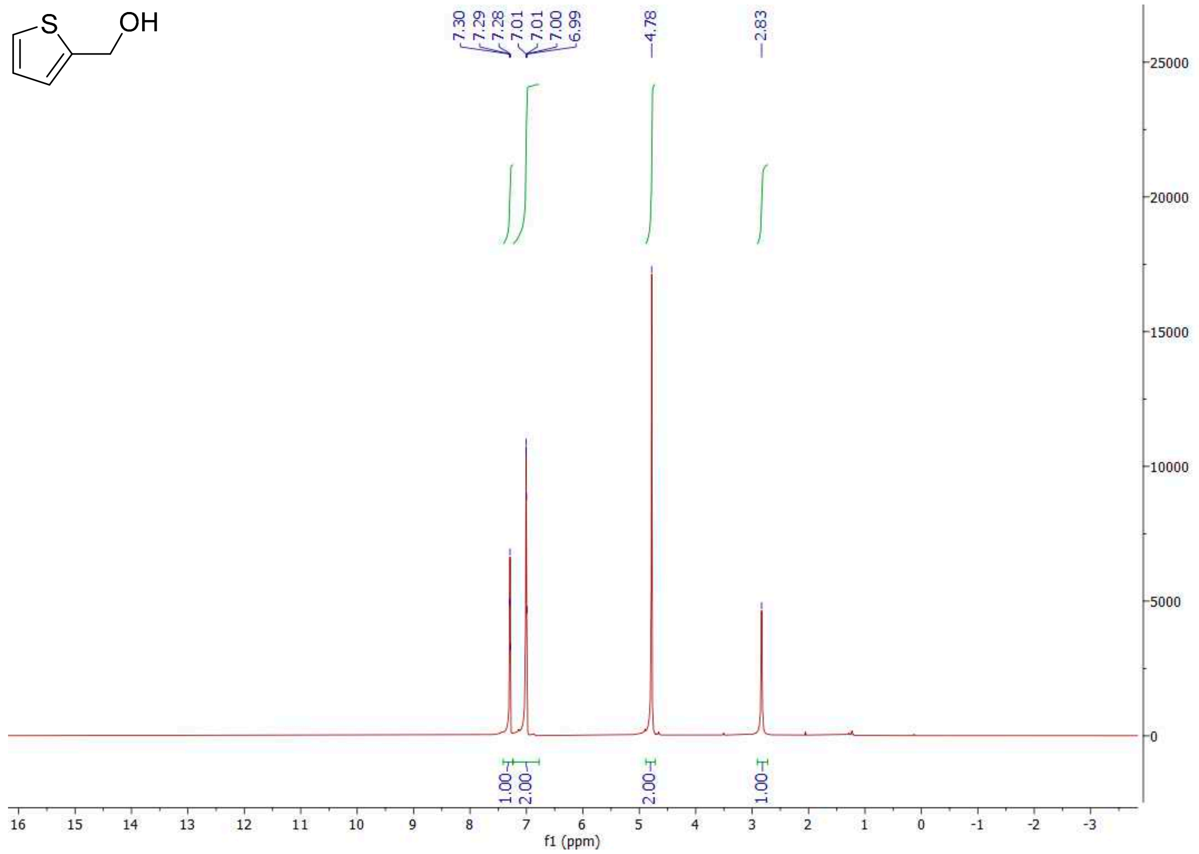

Figure S27. ^{1}H NMR Spectra of thiophen-2-ylmethanol (1n).

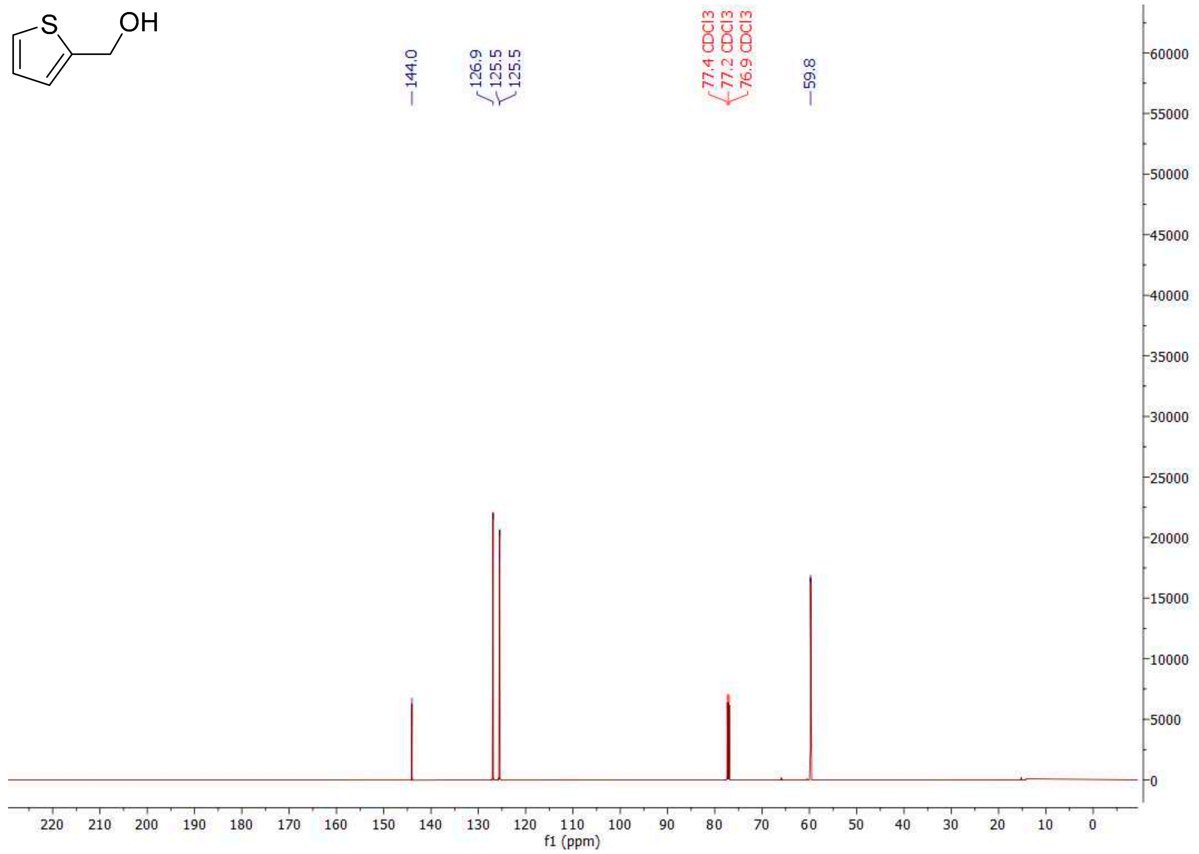

Figure S28. ^{13}C NMR Spectra of thiophen-2-ylmethanol (1n).

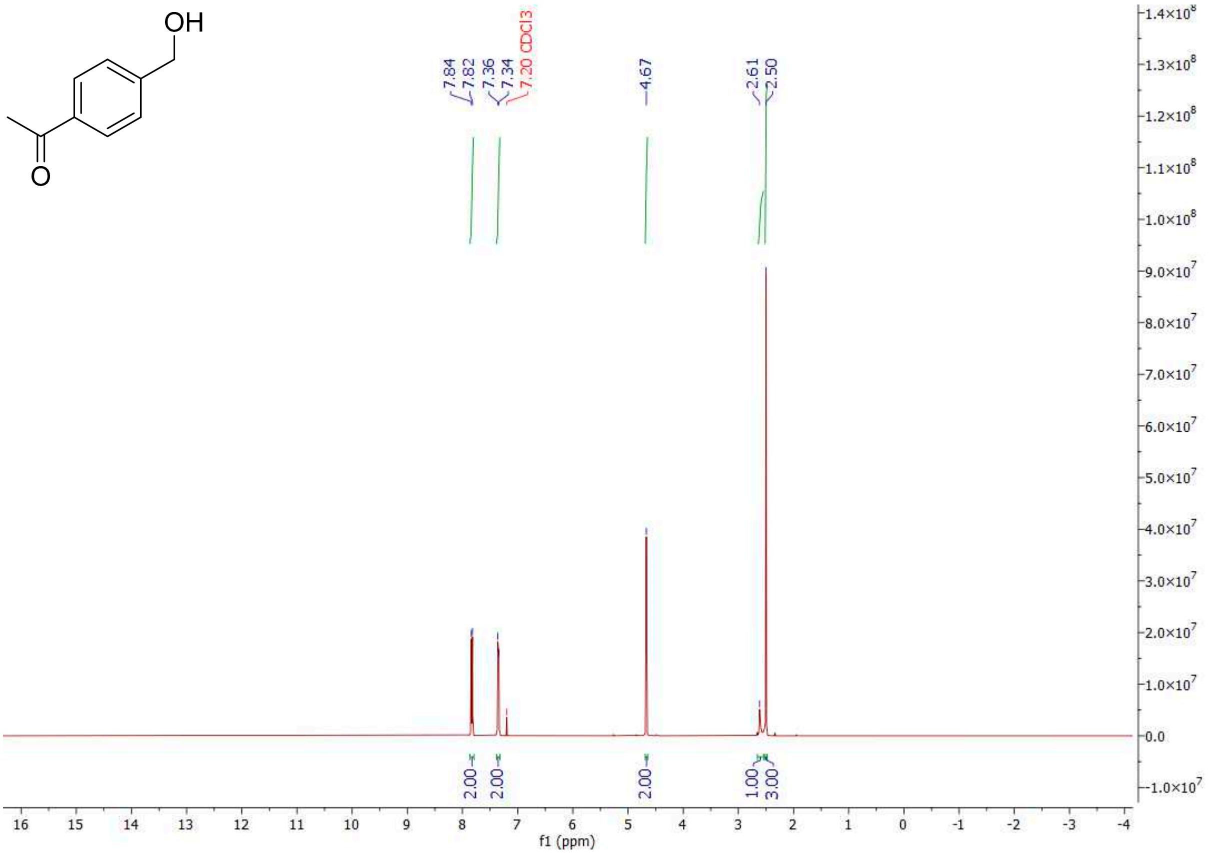

Figure S29. ^{1}H NMR Spectra of 1-(4-(hydroxymethyl)phenyl)ethan-1-one (1o).

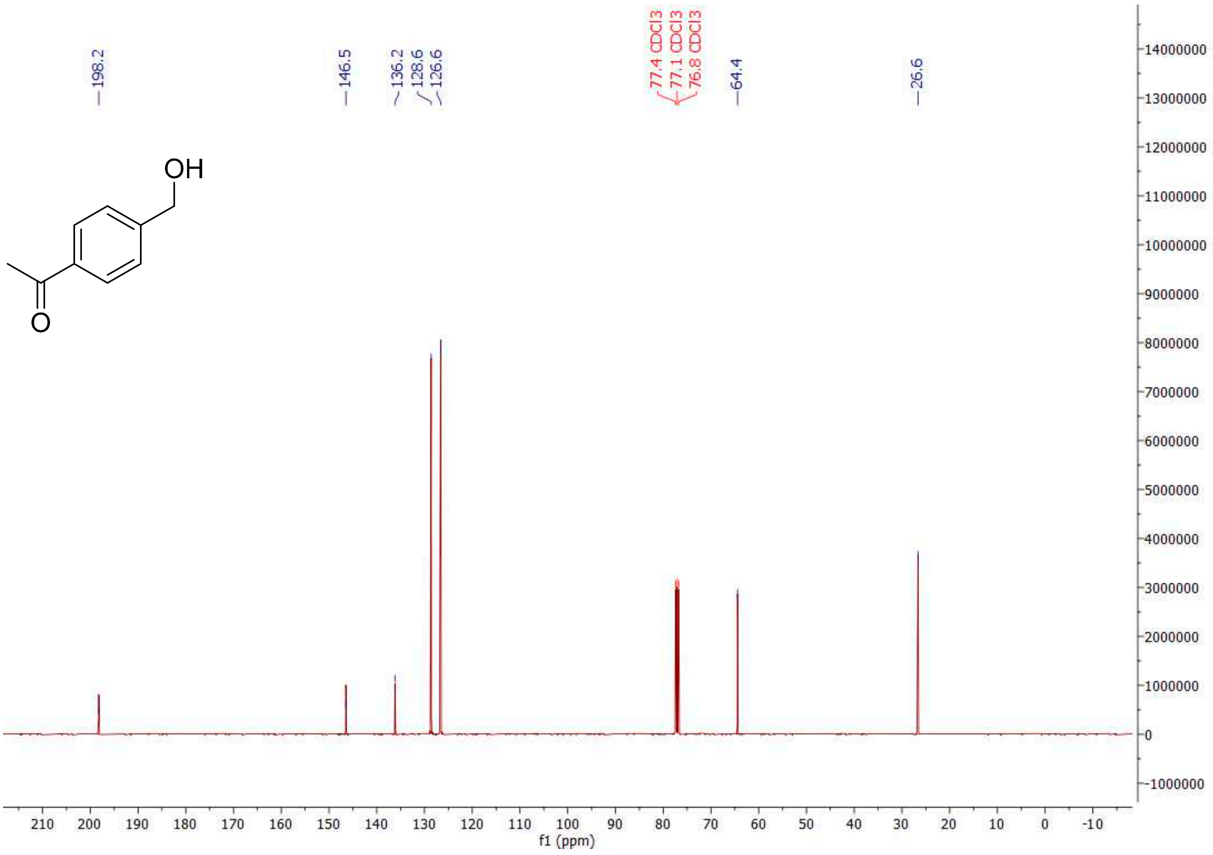

Figure S30. ^{13}C NMR Spectra of 1-(4-(hydroxymethyl)phenyl)ethan-1-one (1o).

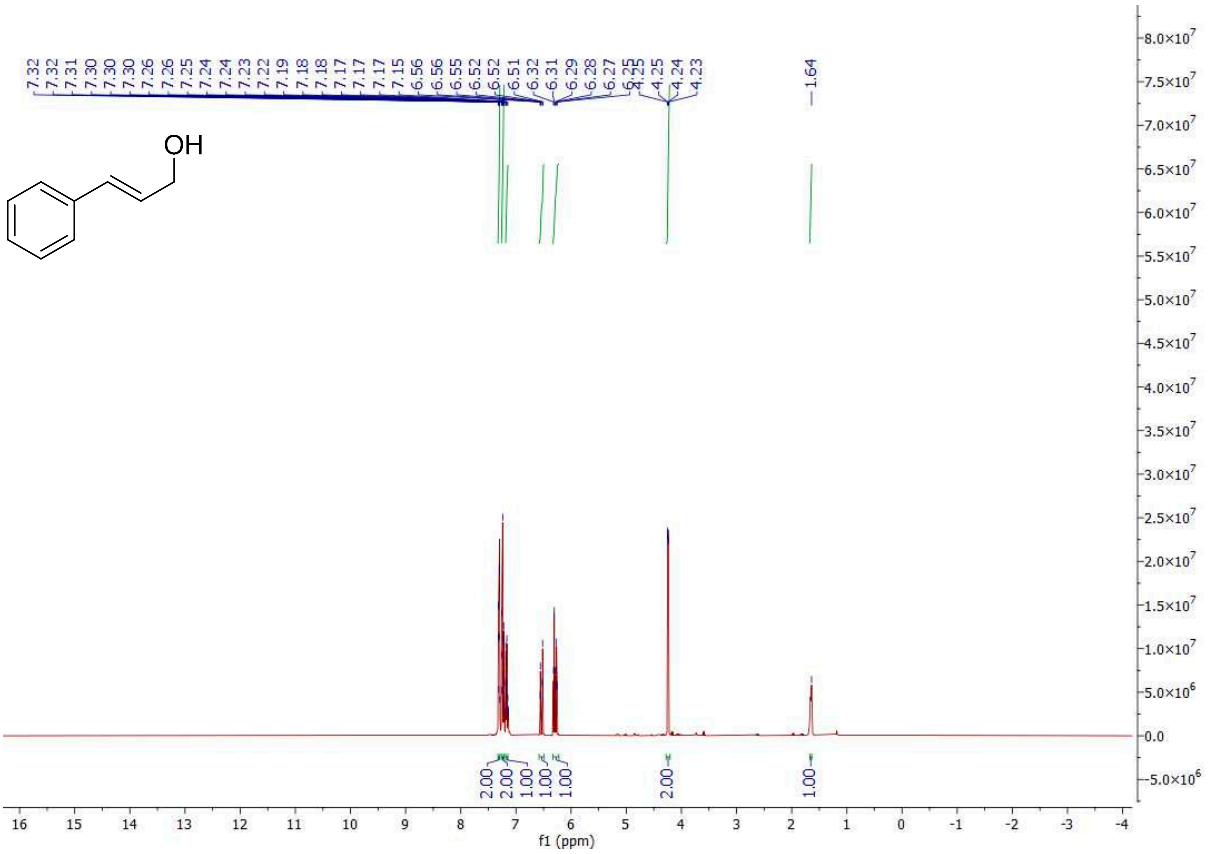

Figure S31. ^{1}H NMR Spectra of (E)-3-phenylprop-2-en-1-ol (1p).

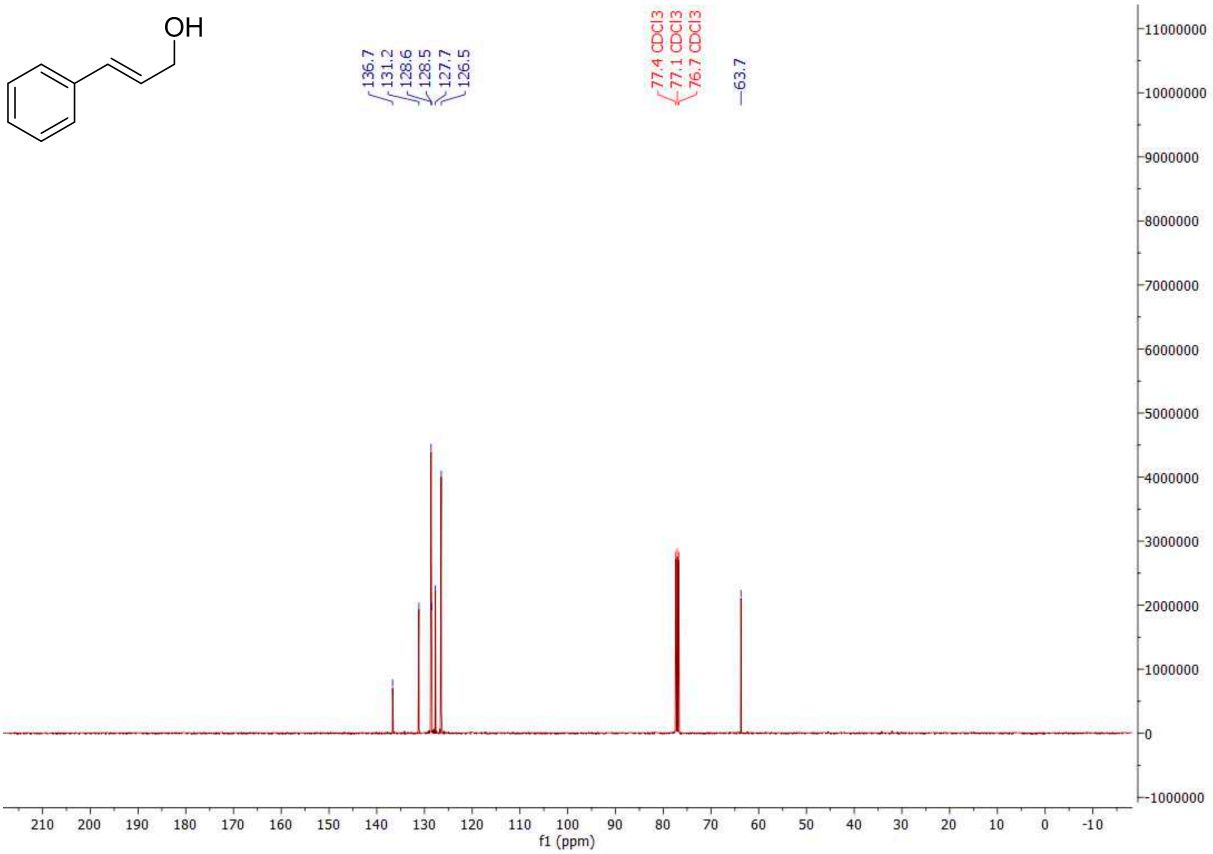

Figure S32. ^{13}C NMR Spectra of (E)-3-phenylprop-2-en-1-ol (1p).

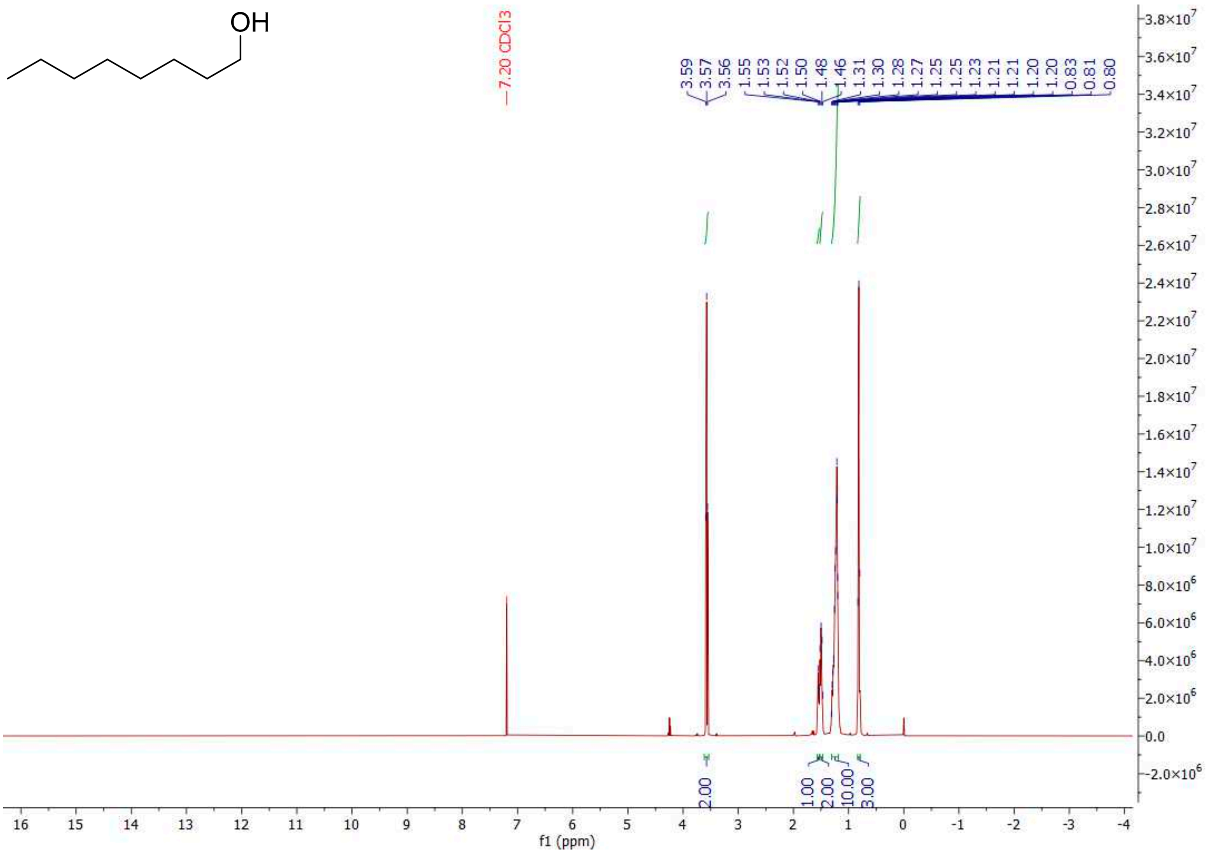

Figure S33. ^{1}H NMR Spectra of octan-1-ol (1r).

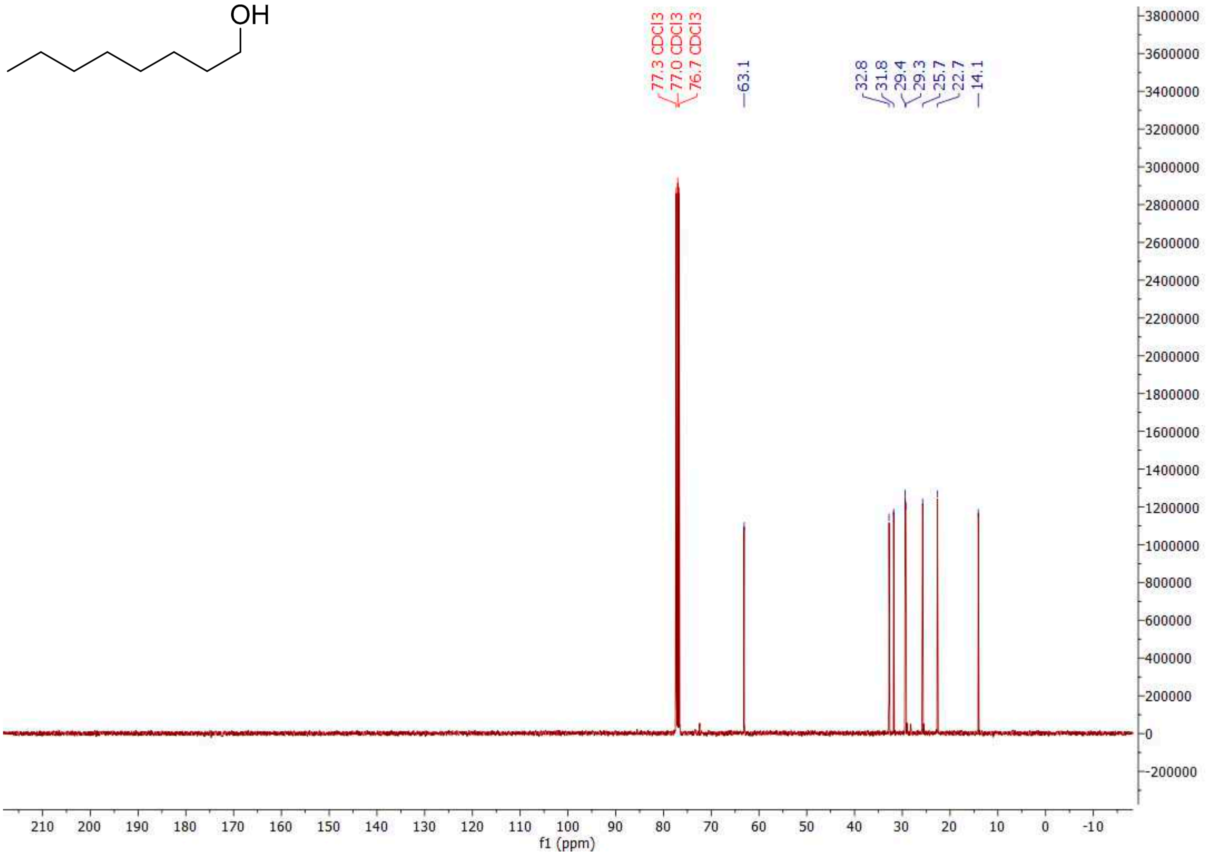

Figure S34. ^{13}C NMR Spectra of octan-1-ol (1r).

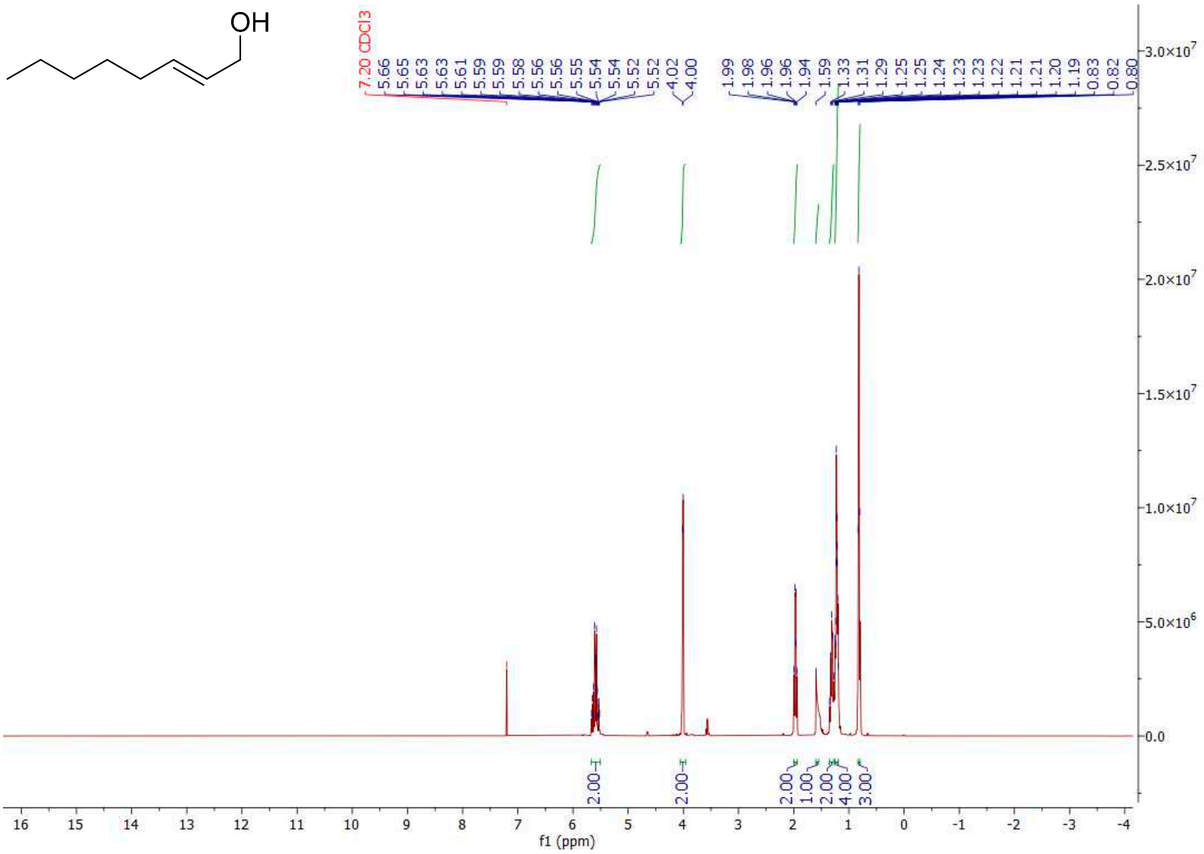

Figure S35. ^{1}H NMR Spectra of (E)-oct-2-en-1-ol (1s).

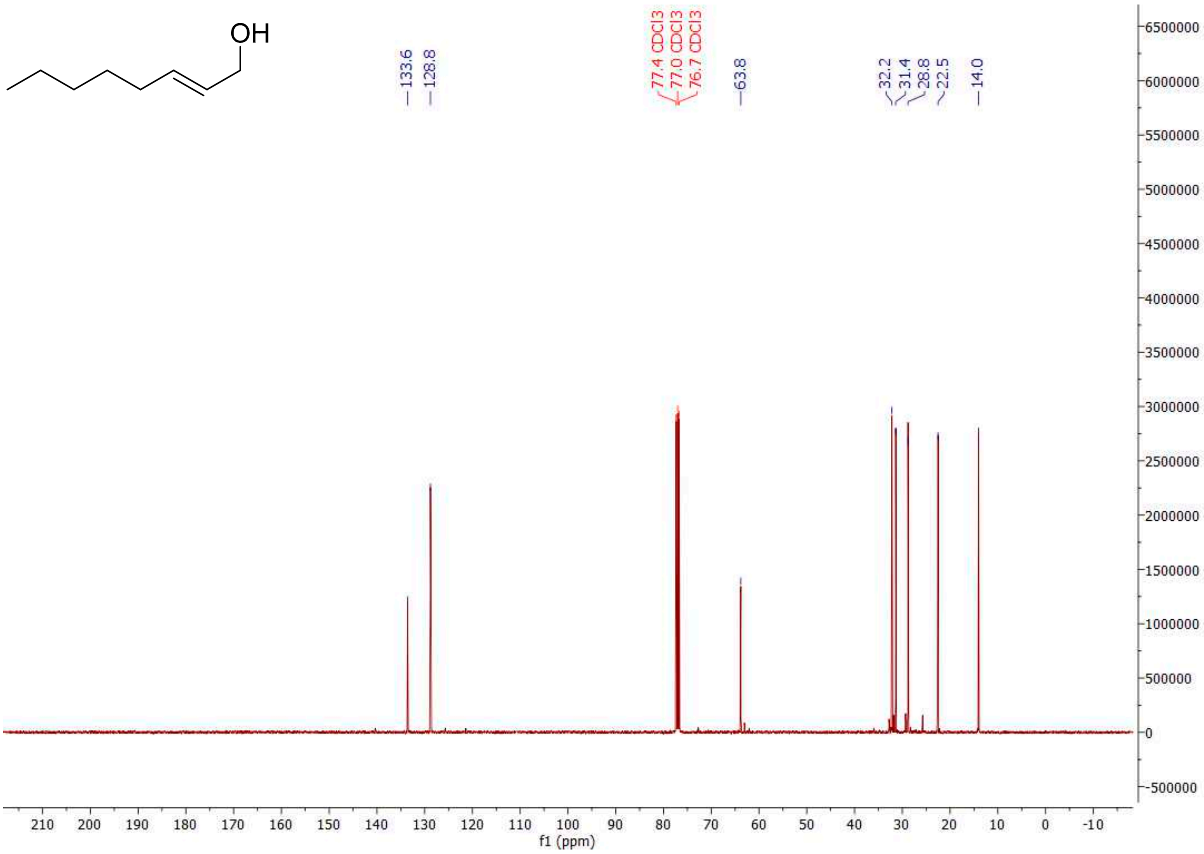

Figure S36. ^{13}C NMR Spectra of (E)-oct-2-en-1-ol (1s).

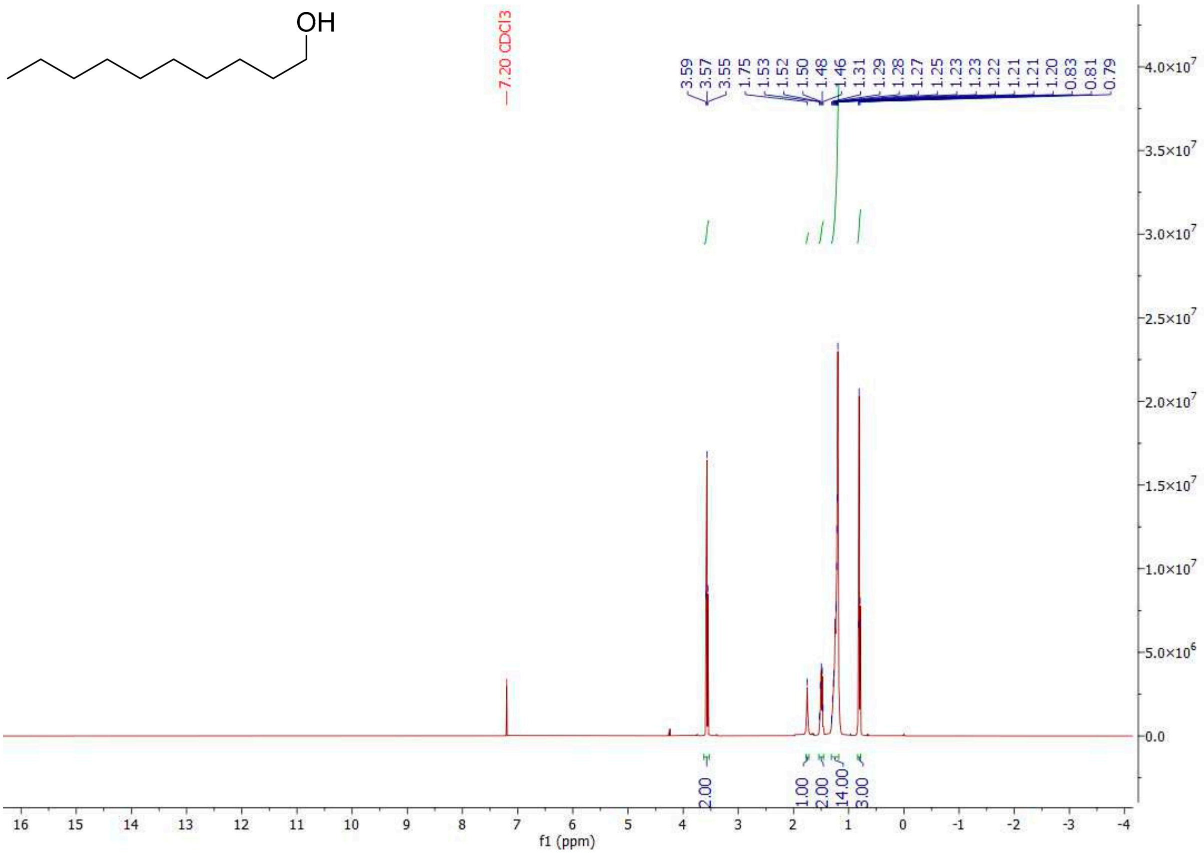

Figure S37. ^{1}H NMR Spectra of decan-1-ol (1t).

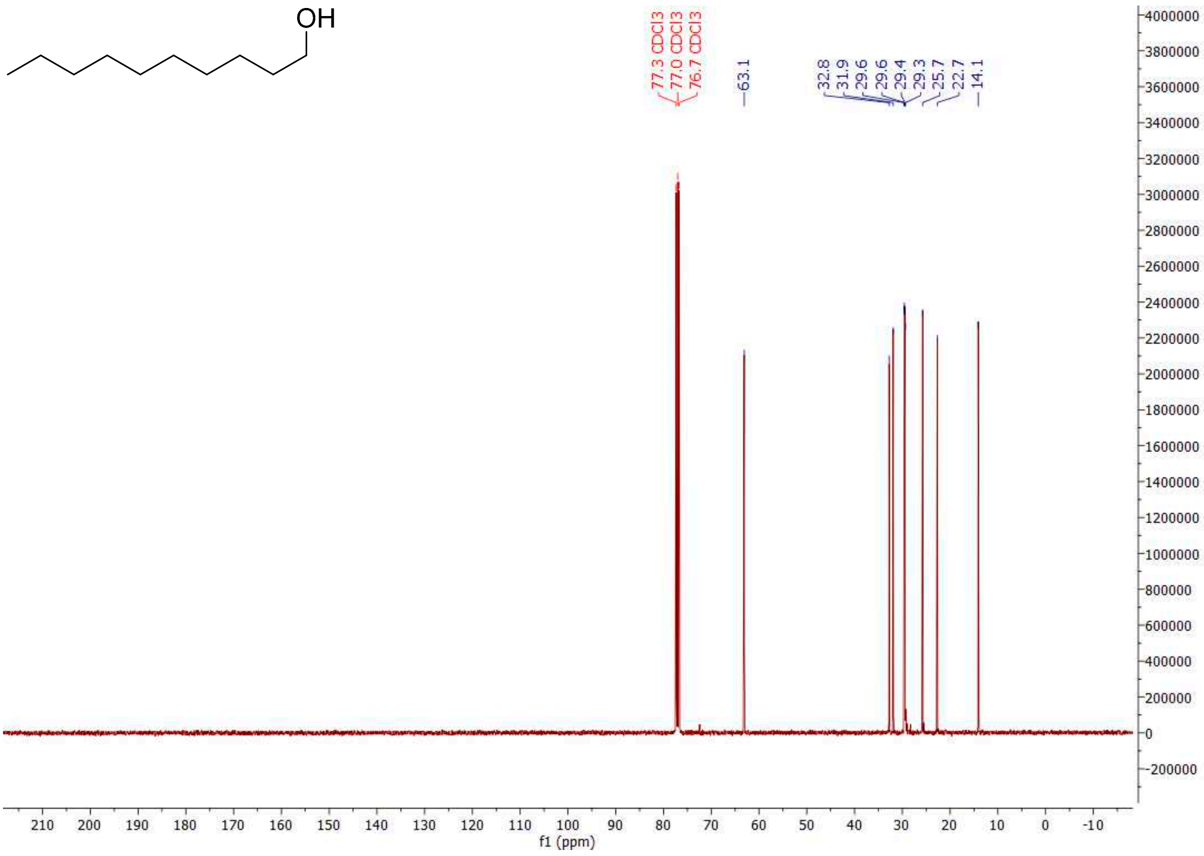

Figure S38. ^{13}C NMR Spectra of decan-1-ol (1t).

6. References

1. Deibl, N.; Kempe, R. General and Mild Cobalt-Catalyzed C-Alkylation of Unactivated Amides and Esters with Alcohols. *J. Am. Chem. Soc.* **2016**, *138*, 10786–10789, doi:10.1021/jacs.6b06448.

2. Sharma, P.K.; Nielsen, P. New Ruthenium-Based Protocol for Cleavage of Terminal Olefins to Primary Alcohols: Improved Synthesis of a Bicyclic Nucleoside. *J. Org. Chem.* **2004**, *69*, 5742–5745, doi:10.1021/jo0491861.

3. Shaikh, N.S.; Junge, K.; Beller, M. A Convenient and General Iron-Catalyzed Hydrosilylation of Aldehydes. *Org. Lett.* **2007**, *9*, 5429–5432, doi:10.1021/ol7021802.

4. Wang, R.; Tang, Y.; Xu, M.; Meng, C.; Li, F. Transfer Hydrogenation of Aldehydes and Ketones with Isopropanol under Neutral Conditions Catalyzed by a Metal-Ligand Bifunctional Catalyst [Cp∗Ir(2,2′-BpyO)(H2O)]. *J. Org. Chem.* **2018**, *83*, 2274–2281, doi:10.1021/acs.joc.7b03174.

5. Griffin, P.R.; Kamenecka, T.M.; Doebelin, C.; Chang, M.R. RORγ Modulators. WO 2018/52903, World Intellectual Property Organization, 2018.

6. Aboo, A.H.; Bennett, E.L.; Deeprose, M.; Robertson, C.M.; Iggo, J.A.; Xiao, J. Methanol as Hydrogen Source: Transfer Hydrogenation of Aromatic Aldehydes with a Rhodacycle. *Chem. Commun.* **2018**, *54*, 11805–11808, doi:10.1039/c8cc06612d.

7. Zanon, J.; Klapars, A.; Buchwald, S.L. Copper-Catalyzed Domino Halide Exchange-Cyanation of Aryl Bromides. *J. Am. Chem. Soc.* **2003**, *125*, 2890–2891, doi:10.1021/ja0299708.

8. Denmark, S.E.; Butler, C.R. Vinylation of Aryl Bromides Using an Inexpensive Vinylpolysiloxane. *Org. Lett.* **2006**, *8*, 63–66, doi:10.1021/ol052517r.

9. Talwar, D.; Wu, X.; Saidi, O.; Salguero, N.P.; Xiao, J. Versatile Iridicycle Catalysts for Highly Efficient and Chemoselective Transfer Hydrogenation of Carbonyl Compounds in Water. *Chem. Eur. J.* **2014**, *20*, 12835–12842, doi:10.1002/chem.201403701.

10. Doni, E.; O'Sullivan, S.; Murphy, J.A. Metal-Free Reductive Cleavage of Benzylic Esters and Ethers: Fragmentations Result from Single and Double Electron Transfers. *Angew. Chem. Int. Ed.* **2013**, *52*, 2239–2242, doi:10.1002/anie.201208066.
